# Endothelial cell‐derived extracellular vesicles induce pro‐angiogenic responses in mesenchymal stem cells

**DOI:** 10.1002/2211-5463.13650

**Published:** 2024-03-29

**Authors:** Hüseyin Abdik, Oğuz Kaan Kırbaş, Batuhan Turhan Bozkurt, Ezgi Avşar Abdik, Taha Bartu Hayal, Fikrettin Şahin, Pakize Neslihan Taşlı

**Affiliations:** ^1^ Department of Molecular Biology and Genetics, Faculty of Engineering and Natural Sciences İstanbul Sabahattin Zaim University Turkey; ^2^ Department of Genetics and Bioengineering, Faculty of Engineering and Architecture Yeditepe University Turkey; ^3^ Department of Aquatic Genomics, Faculty of Aquatic Sciences Istanbul University Turkey

**Keywords:** angiogenesis, differentiation, endothelial cells, exosome, mesenchymal stem cell, small extracellular vesicles

## Abstract

Angiogenesis is a central component of vital biological processes such as wound healing, tissue nourishment, and development. Therefore, angiogenic activities are precisely maintained with secreted factors such as angiopoietin‐1 (Ang1), fibroblast growth factor (FGF), and vascular endothelial growth factor (VEGF). As an element of intracellular communication, extracellular vesicles (EVs)—particularly EVs of vascular origin—could have key functions in maintaining angiogenesis. However, the functions of EVs in the control of angiogenesis have not been fully studied. In this study, human umbilical vein endothelial cell line (HUVEC)‐derived small EVs (<200 nm; HU‐sEVs) were investigated as a potential pro‐angiogenic agent. Treating mesenchymal stem cells (MSCs) and mature HUVEC cells with HU‐sEVs induced their tube formation under *in vitro* conditions and significantly increased the expression of angiogenesis‐related genes, such as *Ang1*, *VEGF*, *Flk‐1* (VEGF receptor 2), *Flt‐1* (VEGF receptor 1), and *vWF* (von Willebrand Factor), in a dose‐dependent manner. These results indicate that HU‐sEVs take part in angiogenesis activities in physiological systems, and suggest endothelial EVs as a potential therapeutic candidate for the treatment of angiogenesis‐related diseases.

## Graphical abstract

Human umbilical vein endothelial cell line‐derived small extracellular vesicles (EVs; <200 nm) (HU‐sEVs) were used as a pro‐angiogenic agent. They were characterized by evaluating morphology, size, and count. Chemical properties of HU‐sEVs were also investigated by antibody‐based and omics. All were based on MISEV criteria. Positive biological functions of HU‐sEVs were proven by differentiation and angiogenesis assays on mesenchymal stem cells and HUVECs.

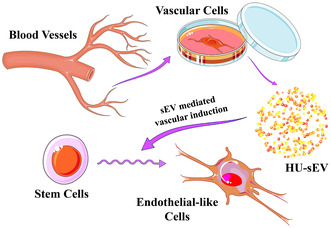

**Abbreviations**
AFM: atomic force microscopy
Ang1: Angiopoietin‐1
ATPS: aqueous two‐phase system
CANX: calnexin
DMEM: Dulbecco's Modified Eagle's Medium
FAME: fatty acid methyl ester
FBS: fetal bovine serum
FGF: fibroblast growth factor
Flk‐1: VEGF receptor 2
Flt‐1: VEGF receptor 1
GC–MS: gas chromatography–mass spectrometry
GO: Gene Ontology
HU‐Cell: HUVEC
HU‐sEVs: human umbilical vein endothelial cell line‐derived small EVs
HUVEC: human umbilical vein endothelial cell
MIS: Microbial Identification System
MSCs: mesenchymal stem cells
MTS: 3‐(4,5‐di‐methyl‐thiazol‐2‐yl)‐5‐(3‐carboxy‐methoxy‐phenyl)‐2‐(4‐sulfo‐phenyl)‐2H‐tetrazolium
NC: negative control
NTA: nanoparticle tracking analysis
PANTHER: Protein Analysis Through Evolutionary Relationships
PBS: phosphate‐buffered saline
PC: positive control
PEG: polyethylene glycol
PSA: penicillin/streptomycin/amphotericin
qPCR: quantitative polymerase chain reaction
SEM: scanning electron microscopy
sEV: small extracellular vesicles
TSG101: tumor susceptibility gene 101
VEGF: vascular endothelial growth factor
vWf: von Willebrand factor

Angiogenesis is the process of vascular growth to provide nourishment to tissues and organs. Angiogenic activities happen throughout the lifespan of an organism, starting with the rapid expansion of the vascular system during embryonic development. After embryonic development, the frequency of angiogenic activities falls significantly and only occurs under tight restrictions in a localized manner [1], such as during the regeneration of wounds and the ovarian cycle [2], and during bone development and fracture healing [3].

An imbalance of angiogenesis is the cause for many pathological conditions. Chronic wounds encountered frequently in diabetes patients are primarily caused by the inadequate formation of neovascularization and dysregulation in angiogenesis activities due to lasting inflammation in endothelial cells and high glucose levels in blood [4, 5]. This disruption prevents the adequate transportation of micronutrients and oxygen to the wound site, which prevents diabetic wounds from healing [6].

Angiogenesis is tightly controlled by several molecular signals. VEGF (vascular growth factor also known as VEGFA) and corresponding receptor proteins such as VEGFR1 (VEGF receptor 1, Flt‐1) and VEGFR2 (VEGF receptor 2, flk‐1) carry out vascular endothelial cell proliferation and angiogenesis [7, 8]. Angiopoietin‐1 (Ang‐1) also displays a crucial function in angiogenesis. It has been demonstrated that a lack of Ang‐1 causes vascular deficiency in mice [9]. In addition, von Willebrand factor (vWF) is a multifunctional glycoprotein, which has a crucial role in homeostasis and regulates angiogenesis [10, 11]. Cells producing these signals may secrete them packed in extracellular vesicles (EVs).

Extracellular vesicles are lipid‐bound vesicles secreted by all cell types. Their primary function is to carry cellular signals and cargo such as proteins, nucleic acids, and other molecules between cells [12]. In addition to transporting molecules between cells, the EV itself may initiate responses at cells that interact with it by binding to cell membrane proteins with its own membrane proteins [13]. Uniquely, the EVs may transfer membrane proteins between cells by fusing their membrane to the recipient cell, effectively joining the vesicle membrane with the cellular membrane of its target [14]. Since their initial discovery, studies have discovered that EVs take an active part in almost all cellular functions. EVs partake in biological functions such as differentiation [15], immune response [16], angiogenesis [17], and pathological functions such as tumorigenesis [18], and immune avoidance [19].

Due to the critical functions of angiogenesis, the discovery of effective pro‐angiogenic agents is crucial and necessary. In this study, human umbilical vein endothelial cell (HUVEC)‐derived small EV (<200 nm) (human umbilical vein endothelial cell line‐derived small EVs, HU‐sEVs) were used as a potential pro‐angiogenic agent. HU‐sEVs were isolated, and their physical, proteomic, and lipidomic profiles were characterized. The effects of HU‐sEVs on the cellular viability, tube formation capacity, and the expression of angiogenesis‐related genes were evaluated with an *in vitro* study and tube formation assays. In addition, sEVs were chosen in this study because they are smaller than 200 nm and are easier to use in tissue engineering.

## Material and methods

### Cell culture conditions

HUVEC (ATCC CRL‐1730) and mesenchymal stem cell (MSC; #PT‐5025, Poietics™ Human Dental Pulp Stem Cells) cells were used for this study. HUVEC cells were cultured in Dulbecco's Modified Eagle's Medium (DMEM, #41966‐029, Invitrogen, Gibco, UK) supplemented with 10% fetal bovine serum (FBS, #10500‐064, Invitrogen, Gibco, UK) and 1% penicillin/streptomycin/amphotericin (PSA, Invitrogen, Gibco, UK). MSC cells were cultured according to the manufacturer's instruction a complete media consisting of Basal Medium (#PT‐4927) with supplements (SingleQuot Kit, #PT‐4514). Incubation conditions of the cells were at 37 °C in a humidified atmosphere with 5% CO_2_.

### Media collection

HUVEC cells were cultured at T‐150 cell culture flasks with Dulbecco's Modified Eagle's Medium (DMEM) containing 10% FBS and 1% penicillin–streptomycin–amphotericin until they reached 80% confluence. Media was discarded, and the cells were washed with phosphate‐buffered saline (PBS) to remove EVs of FBS origin. Cells were then cultured in 30 mL of serum‐free DMEM for 18 h. Media was collected, and cells were given complete media to prevent serum deprivation. Cells were allowed to recover for at least 2 days before another collection. Conditioned media were stored at 20 °C before further use.

### Isolation of HUVEC‐EVs

Human umbilical vein endothelial cell line‐derived small EVs were isolated via aqueous two‐phase system (ATPS) [19] isolation and density cushion ultracentrifugation [20]. ATPS were used in *in vitro* studies, while analytical studies that could have been affected by ATPS polymers were conducted with density cushion ultracentrifugation. For both methods, collected media were first cleared from contaminants including dead cells and cellular debris using differential centrifugation, 300 ***g*** and 2000 ***g*** for 10 min, and 20 000 ***g*** for 30 min, respectively, and were then filtered through a 0.22 μm filter for further removal. For ATPS isolation, resulting supernatants were then mixed at a 1 : 1 v/v ratio with ATPS isolation solution, consisting of 3.35 w/v polyethylene glycol (PEG) (25–45 kDa, Sigma, #81310, St. Louis, MO, USA) and 1.65 w/v dextran (450–650 kDa, Sigma, #81392) in PBS. Two washing solutions were also prepared for each sample, which are 1 : 1 dilution of the ATPS isolation solution with PBS. Samples and their washing solutions were centrifuged at 1000 ***g*** for 10 min for phase separation. Upper phase of the samples, which equates to 80% of their total volume, was then removed and replaced with the upper phase of a washing solution, and was centrifuged at 1000 ***g*** for 10 min for phase separation. The washing procedure was repeated again for each sample with the second set of washing solutions. After the final centrifugation, EV containing bottom phases of the samples (which equate to the 10% of the starting volumes) were extracted.

For density cushion ultracentrifugation, 10 mL of contaminate‐free plasma samples was layered above 1.5 mL of 1 m sucrose solution in a 12.5 mL ultracentrifugation tube. Samples were then centrifuged at 100 000 ***g*** for 80 min using a SW 40i ultracentrifugation rotor (Beckman Coulter, Brea, CA, USA). After the centrifugation, top layer was removed, and 1 mL of the sucrose layer was collected from the bottom to ensure the EV‐containing phase remained unmixed with the contaminants of the upper phase.

### Scanning electron microscopy

Human umbilical vein endothelial cell line‐derived small EVs samples were imagined with a scanning electron microscope to determine their size distribution and morphology. Samples were diluted 1 : 100 and dried on glass microscope slides. Samples were then washed away from excess dextran by dropping 100 μL of chilled methanol to the center of the dried sample, and letting it dry. Samples were coated with gold with a sputter coater (BAL‐TEC SCD 005, Switzerland) and imagine with SEM Zeiss EVO 40 (Jena, Germany).

### Nanoparticle tracking analysis

Size distribution of HU‐sEV and their quantification were done using nanoparticle tracking analysis (Nanosight NS300, Malvern Instruments, Malvern, UK). Samples were diluted to fit the concentrations suggested by the manufacturer. Video capture was done with the low‐volume sample chamber for 60 s at camera level 16. Chamber was flushed with distilled water between each capture. A total of five captures were taken for each sample. Data analysis was done using nanoparticle tracking analysis (nta) software version 3.4.

### Characterization of HU‐sEV surface markers using flow cytometry

Surface markers of HU‐sEV were analyzed using bead‐assisted flow cytometry. HU‐sEV were adhered onto aldehyde/sulfate latex beads (4% w/v, 4 μm, Thermo Fisher, A37304, Waltham, MA, USA). HU‐sEV were incubated with the beads for 15 min at RT on a shaker for proper binding. Bound HU‐sEV were diluted with 200 μL of PBS with 2% BSA to block non‐specific antibody binding. Glycine (Merck, Darmstadt, Germany) was added to the solution to reach 100 mm concentration, incubating for 30 min at RT shaking. Samples were then diluted with 800 μL of cold PBS and centrifuged for 2700 ***g*** for 3 min to pellet the HU‐sEV. Resulting pellet was resuspended with 500 μL of PBS and aliquoted into 100 μL in test tubes and incubated with corresponding antibodies. Markers for CD9 (Biolegend, 124808, San Diego, CA, USA), CD63 (Biolegend 143904), CD81 (Biolegend, 349506), and HSP70 (Biolegend 648004) were at a 1 : 1000 dilution and incubated overnight. Primary antibodies of Alix (Abcam, ab186429, Cambridge, UK), tumor susceptibility gene 101 (TSG101) (Abcam, ab209927), and calnexin (CANX) (Abcam, ab203439) were incubated overnight, centrifuged at 2700 ***g*** for 3 min to wash the samples of excess antibodies and then resuspended in 100 μL of 1 : 100 dilution Alexa Fluor 488 (Abcam, ab150077). Analysis of EVs was done with Becton Dickinson (BD) FACS Calibur Flow Cytometry System (Becton Dickinson, San Jose, CA, USA).

### Capillary western blot

Protein expression profile of HU‐sEV was shown by capillary western blot (Wes, Protein Simple; San Jose, CA, USA). Experimental procedure was carried out according to the manufacturer's instructions. Cell lysate was used as a positive control (PC) to test the functionality of the antibodies used. One to 2 μg total protein was added from cell lysate or Hu‐sEV to the capillary cartridges (12–230 kDa Wes Separation Module 8 × 13 capillary cartridges, Cat#SM‐W002 and 2–40 kDa Wes Separation Module 8 × 13 capillary cartridges, Cat#SM‐W009) to each capillary. Using the Wes system, proteins that correspond with the primary antibodies of Flotillin 1 (D2V7) (1 : 50, Cat#18634), Alix (3A9) (1 : 50, Cat#2171), GM130 (D6B1) (1 : 50, Cat#12480) (Cell Signalling Technology; Denver, MA, USA), CD9 (1 : 50, Cat#10626D), CD81 (1 : 50, Cat#10630D) (Thermo Fisher Scientific; USA) and HRP conjugated Serum Albumin (1 : 50, Cat#ab18193) (Abcam) were detected automatically. Secondary antibodies used were Anti‐Rabbit IgG, HRP‐linked (1 : 1000, Cat#7074) and Anti‐Mouse IgG, HRP‐linked (1 : 1000, Cat#7076) (Cell Signalling Technology; Denver, MA, USA). Calculation and analysis of protein expression were based on the gel‐like images produced by the Compass for sw software (Version 4.0, Protein Simple).

### Atomic force microscopy analysis

Visual imaging of the purified EV samples was performed with atomic power microscopy. While preparing EV samples, they were diluted 1 : 100 with 18 megohm deionized water and dried overnight on an ultra‐flat silicon surface. Subsequently, the prepared samples were scanned using an aluminum‐coated cantilever (NSC36‐B) in contact mode in the Park System XE‐100 atomic force microscopy (AFM). Topographic scanning in EV samples was taken at a scan rate of 2 Hz and a resolution of 256 × 256 pixels. The acquired images were analyzed via xei software (Park Systems, Santa Clara, CA, USA).

### Fatty acid methyl ester assay

Fatty acid profiling of EVs purified from HUVEC cells was performed by fatty acid methyl ester (FAME) analysis. In the analysis, primarily, the total fatty acids of the EVs were isolated and then they were converted to FAME derivatives by transesterification reaction. Samples were prepared according to the manufacturer's instructions. Analysis of prepared samples and identification of fatty acids were performed with Agilent Tech GC‐Midi 6890 N device. In the last stage of the sample preparation process, the upper phase was transferred to GC vial and inserted for gas chromatography–mass spectrometry (GC–MS) analysis. Fatty acid peaks in chromatogram were identified in the Sherlock Database using the Sherlock Microbial Identification System (MIS).

### Proteomic profiling

Proteomic profiles of HU‐sEVs were characterized using mass spectroscopy. Briefly, HU‐sEVs were resuspended in SDS‐PAGE sample buffer, and protein concentrations were measured by performing a Bradford assay (Bio‐Rad, Foster City, CA, USA), measured by a Nanodrop 1000 Spectrophotometer (Thermo Fisher). 12% SDS‐PAGE gel electrophoresis was used for protein separation. Separated proteins were precipitated and concentrated using ReadyPrep 2‐DE Cleanup Kit (Bio‐Rad) per the manufacturer's instructions. Proteins were fixed overnight in a fixation solution (40% methanol, 10% acetic acid, colloidal Coomassie Brilliant Blue G‐250). Proteins were then recovered from the gels using an in‐gel tryptic digestion kit (Thermo Fisher). Analysis of the digested peptides was performed with an nLC‐MS/MS using an Ultimate 3000 RSLC nanosystem (Dionex, Thermo Fisher) coupled with a Q Exactive mass spectrophotometer (Thermo Fisher). MS spectra were obtained with the following settings: resolution of 70.000, scan range of 40–2000 m/z, spray voltage of 2.3 kV, target automatic gain control of ‘AGC’ 3 × 10^6^, and maximum injection time of 60 ms. The top 10 precursor ions were selected by data‐dependent acquisition for MS/MS analysis. Protein candidates were identified using proteome discoverer 2.2 (Thermo Fisher). Resulting proteins were queried against the Uniprot/Swissprot database for identification.

### Cell viability assay

MTS (3‐(4,5‐di‐methyl‐thiazol‐2‐yl)‐5‐(3‐carboxy‐methoxy‐phenyl)‐2‐(4‐sulfo‐phenyl)‐2H‐tetrazolium) (#G3582, CellTiter96 AqueousOne Solution; Promega, Southampton, UK) assay was done to measure the effects of HU‐sEV on cell viability of MSC at Days 1, 5, and 10. MSCs were seeded onto 96‐well plates at a concentration of 5 × 10^3^ cells/well. The next day, HU‐sEV were applied onto cells with various particle numbers including 2.50E+09, 5.00E+09, 10.00E+09, 15.00E+09, 20.00E+09, and 25.00E+09 np·mL^−1^. After each incubation time point (Days 1, 5, and 10), 10 μL MTS solution, and 100 μL PBS/glucose solution onto each well. The plate was incubated for 1 h at 37 °C. Absorbance (nm) was measured at 495 with an ELISA plate reader (Biotek, Winooski, VT). Experiments were carried out both technically and biologically with at least three replicates.

### MSC differentiation using HU‐sEV

Mesenchymal stem cell cells were seeded on 6‐well plates at a number of 5 × 10^4^ cells/well as three replicates for each group. After 24 h, morphology of the cells was checked, and the differentiation process was started. Three different concentrations of HU‐sEV (2.50E+09, 5.00E+09, and 10.00E+09 np·mL^−1^) were prepared in MSC culture media to differentiate MSC cells. Apart from the treatment groups, MSC incubated in culture media was used as a negative control (NC) of differentiation, and endothelial HUVEC cells were used as a positive control of differentiation. Three different concentrations of HU‐sEV containing differentiation media were replenished every other day to MSC cells for 10 days. At the end of the 10th day, cells were collected to confirm and measure differentiation levels of HU‐sEV‐induced MSC.

### Quantitative polymerase chain reaction

HU‐sEV‐treated MSCs were collected to total RNA isolation. Then, cDNAs were synthesized from the RNA for use as a template in PCR technique. To evaluate the levels of angiogenesis‐related markers in the HU‐sEV treated cells, quantitative polymerase chain reaction (qPCR) was done. As markers, Ang1, VEGF, Flt1, Flk1, and vWF were selected (Table 1). HUVEC was used as a PC. SYBR Green (Applied Biosystem, Waltham, MA, USA) was used for carrying out the reaction. iCycler qPCR system (Bio‐Rad, CFX Real Time System, Singapore) was run for analysis. Experiments were carried out both technically and biologically with at least three replicates.

**Table 1:** qPCR primer sequences. Flt 1, VEGFR1; Flk 1, VEGFR2.

Primer	Sequences 5′–3′
Ang 1	CATTCTTCGCTGCCATTCTG
	GCACATTGCCCATGTTGAATC
VEGF	TTGCCTTGCTGCTCTACCTCCA
	GATGGCAGTAGCTGCGCTGATA
Flt 1	GAGGAGGATGAGGGTGTCTATAGGT
	GTGATCAGCTCCAGGTTTGACTT
Flk 1	GCCCTGCTGTGGTCTCACTAC
	CAAAGCATTGCCCATTCGAT
vWF	CCTTGAATCCCAGTGACCCTGA
	GGTTCCGAGATGTCCTCCACAT
18S RNA	CGGCTACCACATCCAAGGAA
	GCTGGAATTACCGCGGCT

### Tube formation assay

Tube formation assay was used as a model experiment for evaluating angiogenesis capacity of the cells under tested agent. HUVEC cells were cultured onto the 150 μL Matrigel‐coated 24‐well plate at a concentration of 1 × 10^5^ cells/well. Three different particle numbers of the HU‐sEV were tested on HUVEC cells. After 7 h, tube‐like structures were qualified and quantified in the aspect of different points including tube length, total loops, and branching points with Wimasis, 2016. wimtube: Tube Formation Assay Image Analysis Solution. Release 4.0. Similarly, to HUVECs, MSCs were seeded onto 150 μL Matrigel‐coated 24‐well plates at a concentration of 1 × 10^5^ cells/well and then treated with HU‐sEV.

### Bioinformatics analysis

**Fig. 1:**
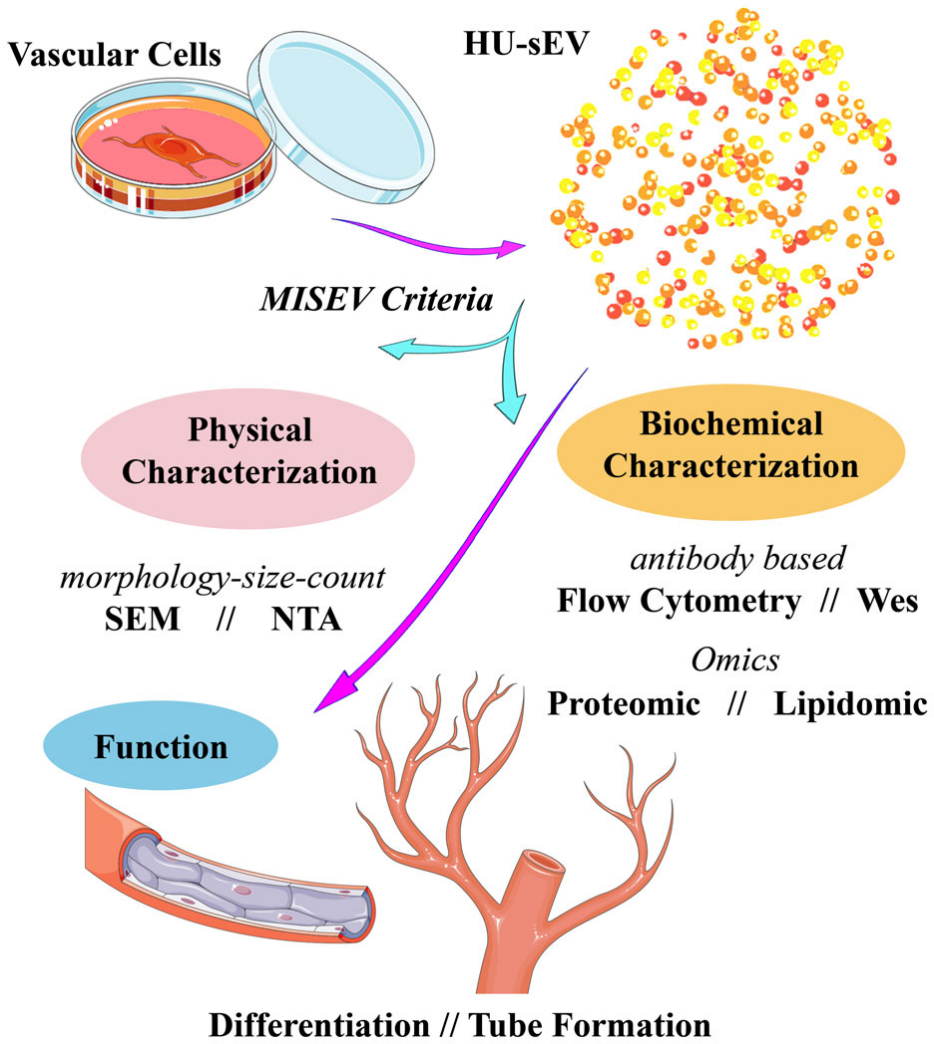
Graphical demonstration of HU‐sEV characterization according to MISEV criteria.

Gene Ontology (GO) enrichment analysis of the HU‐sEV proteome in Fig. 4 was performed using Protein Analysis Through Evolutionary Relationships [21] (PANTHER), using the Fisher's exact test with Bonferroni correction. Fold enrichment of proteins was calculated using the *Homo sapiens* reference database. Percentage of identified proteins was calculated by dividing the number of proteins categorized under a particular GO term with the number of uniquely mapped IDs. Protein–protein associations of the HU‐sEV proteome were visualized using string [22] and cytoscape [23] software. Parts of the Graphical Abstract and Fig. 1 were drawn by using pictures from Servier Medical Art. Servier Medical Art by Servier is licensed under a Creative Commons Attribution 3.0 Unported License.

### Statistical analysis

All data for Figs 4, 5, 6, 7, 8 were statistically analyzed by one‐way ANOVA and Tukey's *post hoc* test using graphpad prism version 8.0.0 for Windows, GraphPad Software, San Diego, California USA, www.graphpad.com. The values of *P < 0.05, **P < 0.01, and ***P < 0.001 were accepted as significant.

## Results

### Physical and biochemical characterization of HU‐sEVs

**Fig. 2:**
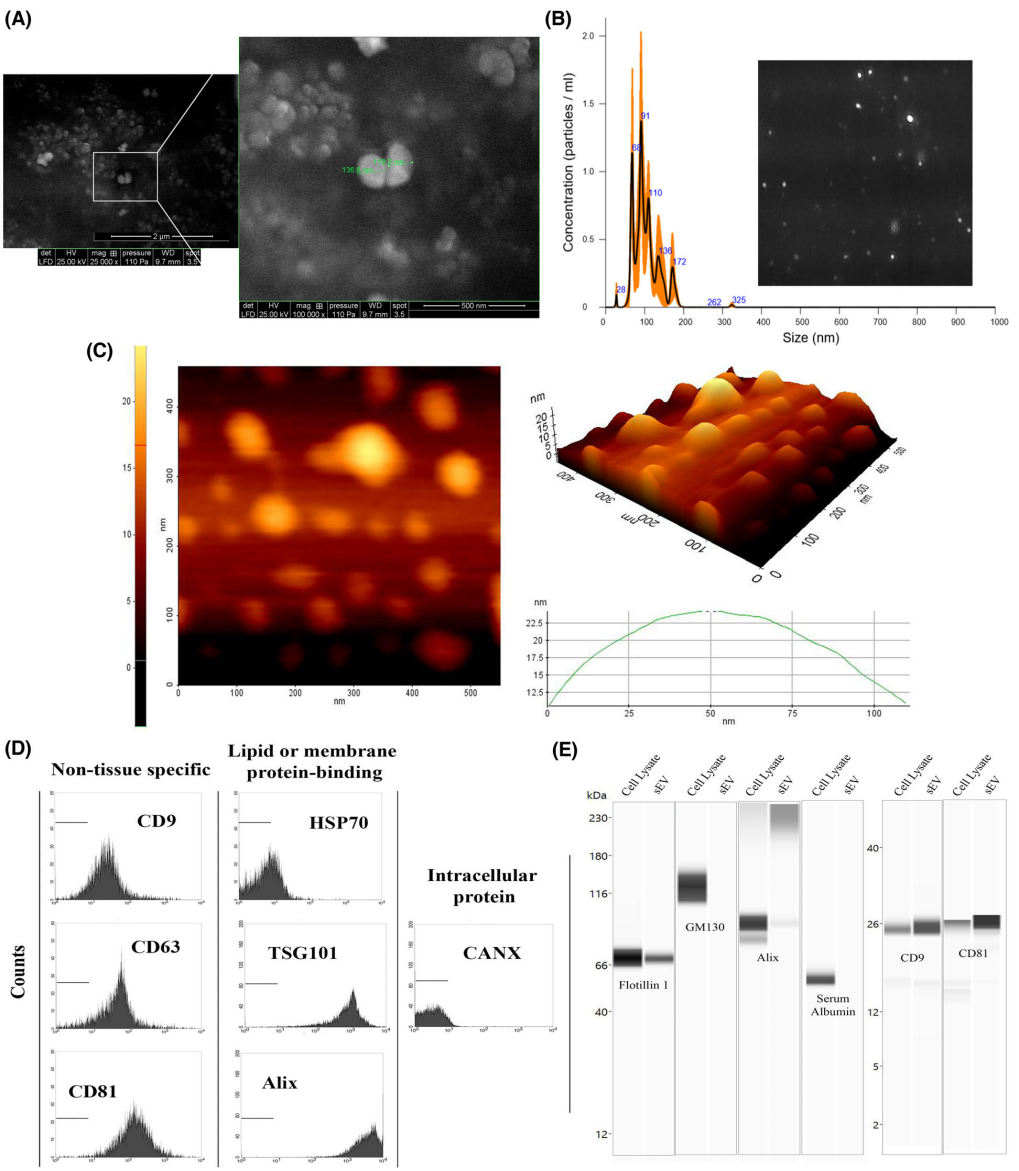
Characterization of HU‐sEV. (A) SEM micrographs of HU‐sEVs. (B) Size distribution of HU‐sEVs with NTA and Brownian motion image. (C) AFM image. Analysis of EV biochemical characterization of HU‐sEVs with (D) flow cytometry, and (E) Capillary Western Blotting (Wes).

Extracellular vesicles isolated from HUVEC cells were characterized according to the standards presented in MISEV2018 [24] (Fig. 1). NTA measurements of HU‐sEVs showed a polydisperse population that was <200 nm in diameter. Particle diameters measured from the E‐SEM micrographs correlated to the NTA measurements (Fig. 2A,B). Besides, the morphological characteristics of HU‐sEVs were analyzed by AFM (Fig. 2C). According to the microscopic images, the sphere‐like shape of HU‐sEVs was observed favorably to inherent features of EVs. Flow cytometry of HU‐sEVs show that they positively express transmembrane proteins CD9, CD63, and CD81; and membrane binding proteins HSP70, TSG101, and Alix (Fig. 2D). Western blots of HU‐sEVs confirmed the presence of CD9, CD81, and Alix. Western blots further show that proteins GM130 and serum albumin, which are used as a control against protein contaminants for EV isolations, were not present in HU‐sEV isolations (Fig. 2E).

**Fig. 3:**
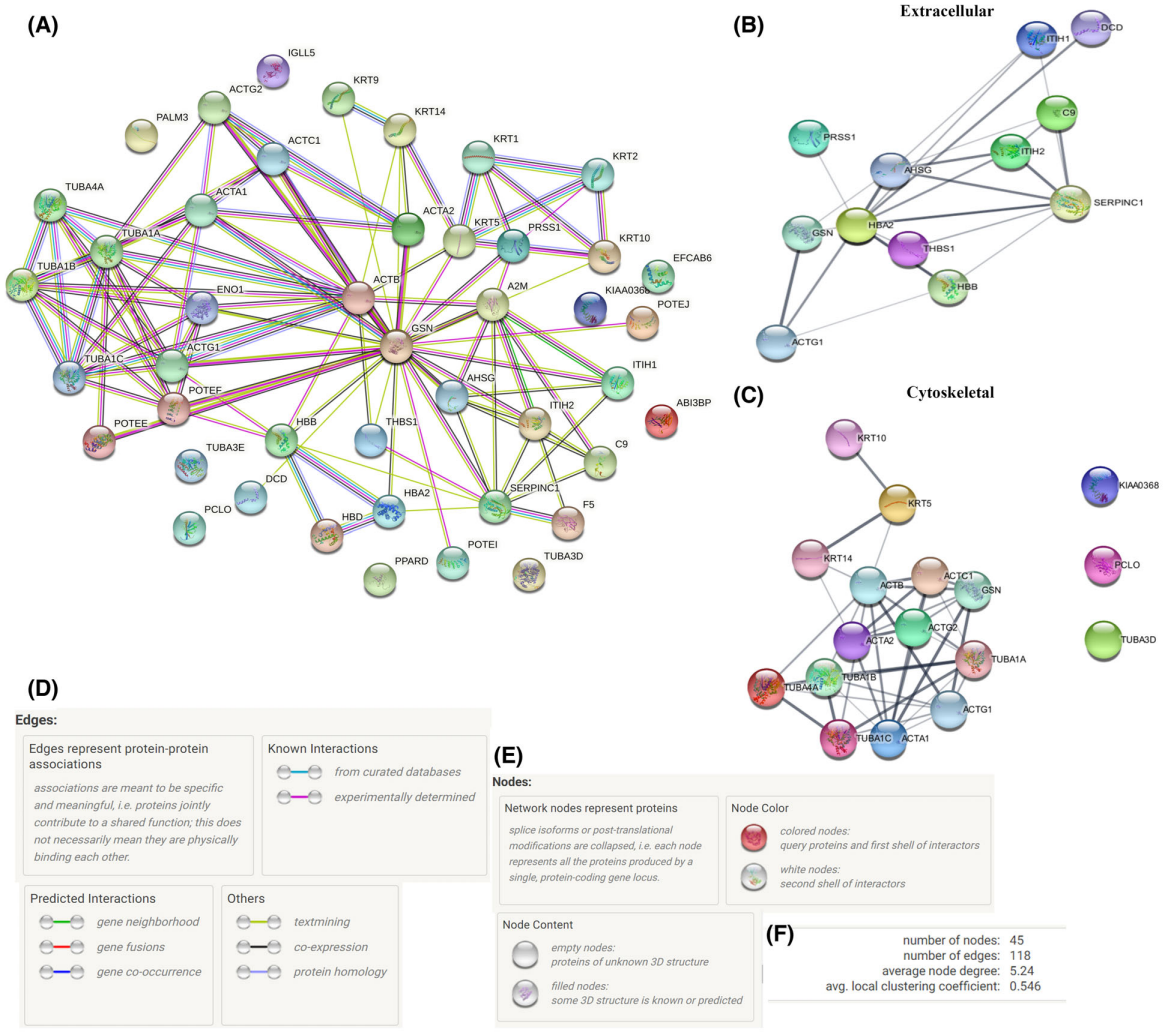
Proteomic analysis of HU‐sEVs. STRING relation scheme of HU‐sEV (A) total proteins, (B) extracellular, and (C) cytoskeletal compartment‐related proteins. (D) Protein–protein associations were represented in the Edges. (E) Significant proteins, splice isoforms, or post‐translational modifications were represented as Network nodes and (F) the network stats related to these interactions were represented.

We performed network interaction analysis for HU‐sEV proteins (Table S1) using string (Fig. 3) and kegg (Table S2) softwares. According to proteomic analysis, the association diagram of these proteins with each other and with the relevant pathways was conducted (Fig. 3A). Additionally, diagrams of enriched proteins serving in ‘Extracellular’ (Fig. 3B) and ‘Cytoskeletal’ (Fig. 3C) compartments were shown. The relevant information about the interactions of enriched protein groups are given in Fig. 3D,E. Besides, network statistics of these interactions also were shown in Fig. 3F.

**Fig. 4:**
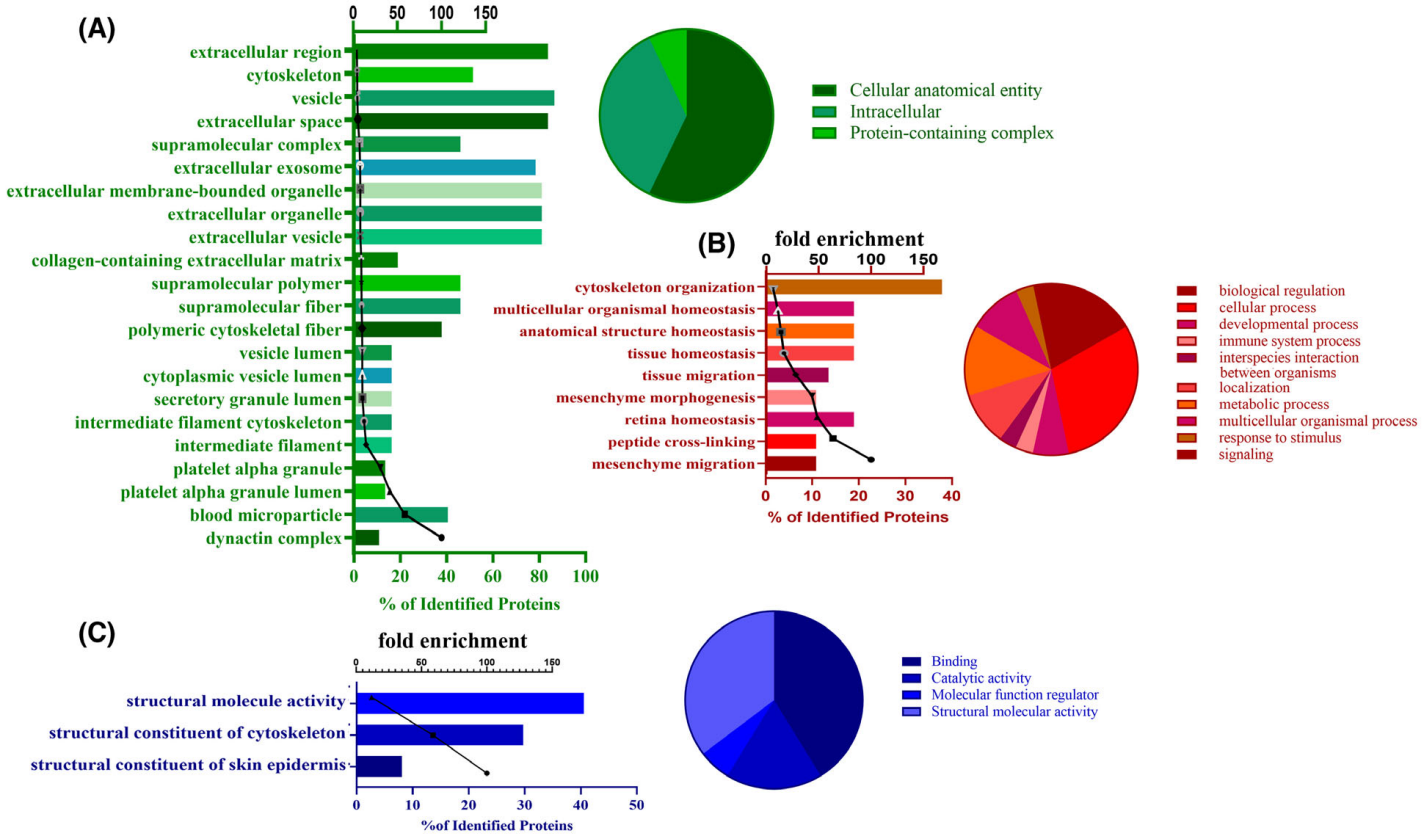
Gene Ontology of HU‐sEV according to functional enrichment networks, (A) cellular component (green), (B) molecular function (red), and (C) biological process (blue). Bars represent the percentage of identified protein for each GO term, while the line graph indicates fold enrichment of said terms compared with the reference human proteome. Pie charts show the percentage of identified proteins for the terms with the highest hierarchical orders.

Gene Ontology enrichment profiles of EV proteomes can be useful in understanding the nature and biological functions of the EV (Fig. 4). HU‐sEVs were compared with the human reference background, which represent the average expression levels of different protein groups (Fig. 4A). Analyses revealed that HU‐sEVs were enriched in proteins under categories such as ‘mesenchyme migration’, ‘mesenchyme morphogenesis’, and ‘tissue migration’. Of the identified proteins, 37.8% were related to cytoskeleton organization (Fig. 4B). Furthermore, around 83–78% of the proteins were annotated under ‘vesicle’, ‘extracellular exosome’, ‘extracellular vesicle’, and similar categories. Molecular functions of the HU‐sEV proteins were primarily structural (Fig. 4C).

**Fig. 5:**
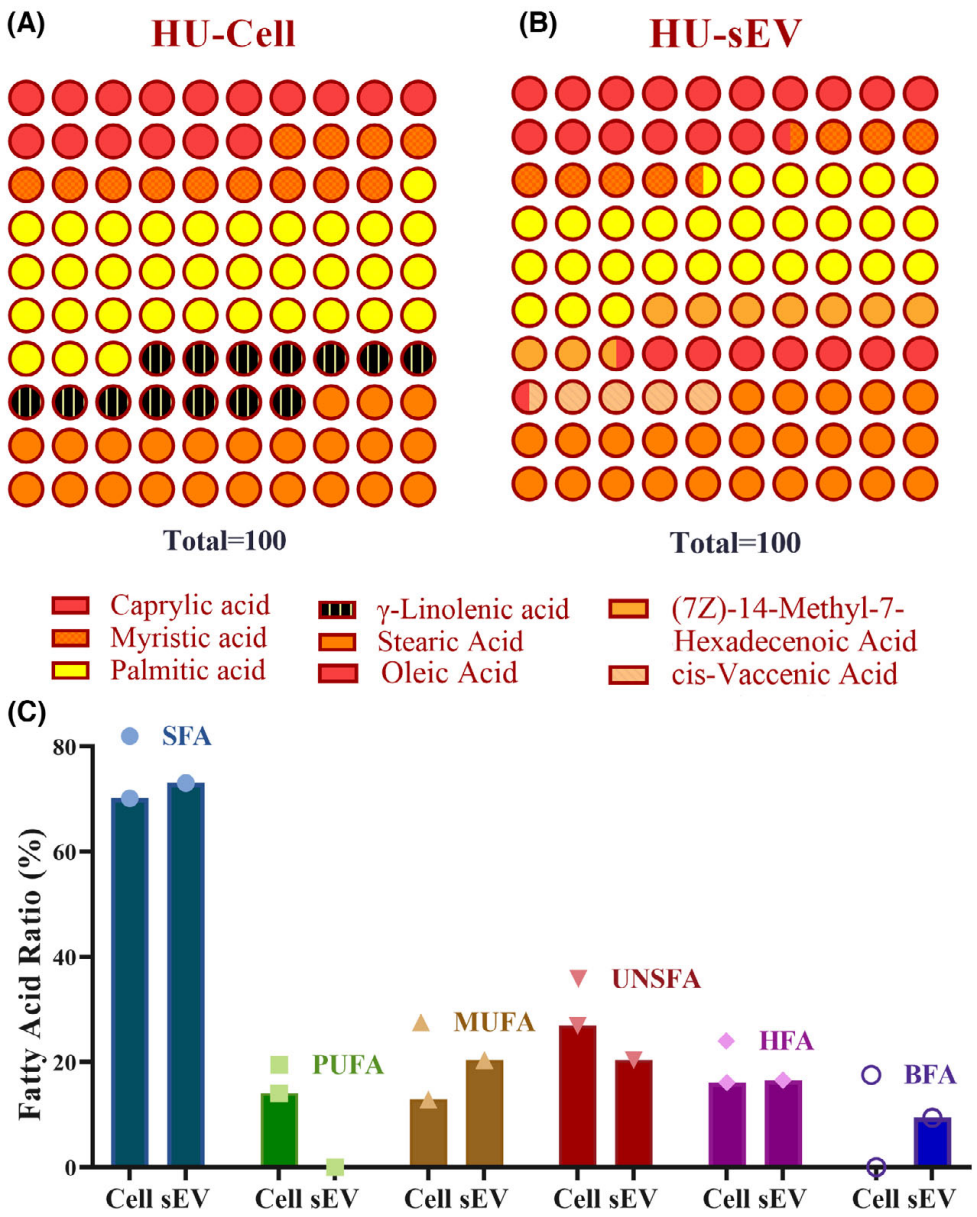
Comparative FAME results. Quantitative results of fatty acid content in (A) HU‐Cell and (B) HU‐sEV samples. (C) Fatty acid ratio of HU‐Cell and HU‐sEV samples. BFA, branched fatty acid; HFA, hydroxy fatty acid; MUFA, mono‐unsaturated fatty acid; PUFA, polyunsaturated fatty acid; SFA, saturated fatty acid; UNSFA, unsaturated fatty acid.

To identify fatty acid content expressed from HUVEC cell pellet and HU‐sEV, three replicate samples were analyzed by gc‐fame software (Fig. 5). Fatty acid ratios obtained from HU‐Cell (HUVEC) GC‐FAME analysis were detected and the ratios are caprylic acid 16.59%, myristic acid 7.72%, palmitic acid 28.78%, (7Z)‐14‐methyl‐7‐hexadecenoic acid 9.44%, oleic acid 8.08%, cis‐vaccenic acid 4.58%, and stearic acid 24.8% (Fig. 5A). HU‐sEV fatty acid ratios are caprylic acid 16.1%, myristic acid 12.9%, palmitic acid 34.2%, γ‐linolenic acid 14.05%, and stearic acid 22.75% (Fig. 5B). In addition, fatty acid distribution was determined according to the saturation and other modification properties of fatty acids (Fig. 5C).

### Effects of HU‐sEV on morphology and cell viability

**Fig. 6:**
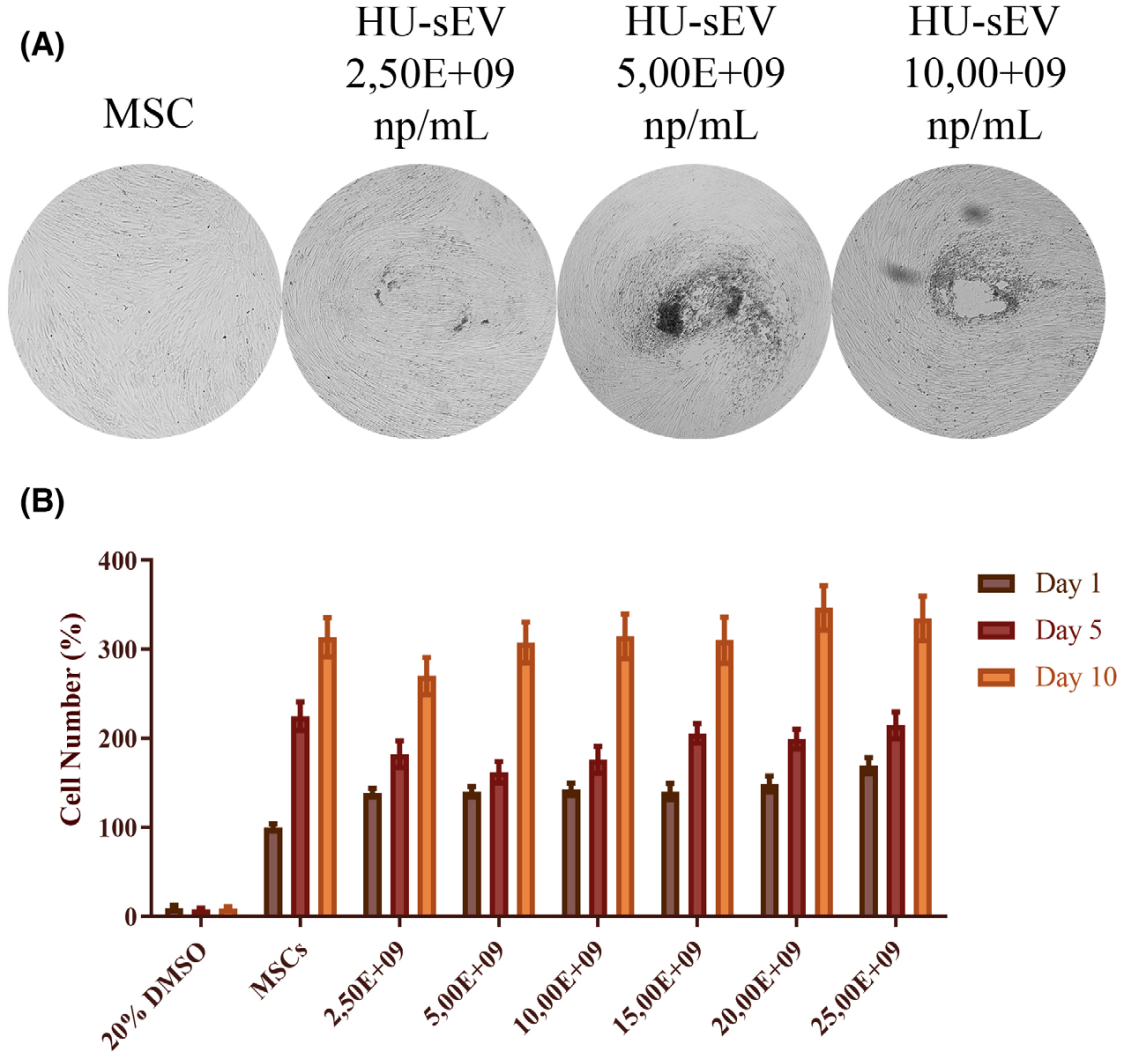
Effect of HU‐sEV on MSC cell morphology and cell viability. (A) HU‐sEV‐treated cells formed a round structure as the dose increased and gained tubular shape, qualitative analysis evaluated by bright field 5× magnification light microscope images. (B) Graphical demonstration of cell viability of HU‐sEV‐treated MSCs in a dose‐dependent manner at 1st, 5th, and 10th days. Values are reported as the means ± SD.

During angiogenesis, mesenchymal stem cells (MSCs) form capillary tube‐like structures. To study their pro‐angiogenic potentials, various concentrations of HU‐sEVs were added to MSC cultures, and the cultures were observed for any morphological changes over a 10‐day period. Over this duration, cells in groups treated with HU‐sEVs, and not the control groups, exhibited morphological changes that lead to the formation of circular intercellular spaces reminiscent of tubes. The frequency and degree of the morphological changes were dose dependent of HU‐sEVs, and increased with increasing concentrations (Fig. 6A). A cell viability experiment was performed to determine the nontoxic doses of HU‐sEVs to be used in the differentiation assay. HU‐sEVs did not display any cytotoxic or proliferative effects on MSCs for the tested concentrations (Fig. 6B).

### HU‐sEV can induce tube formation capacities of MSCs and HUVEC

**Fig. 7:**
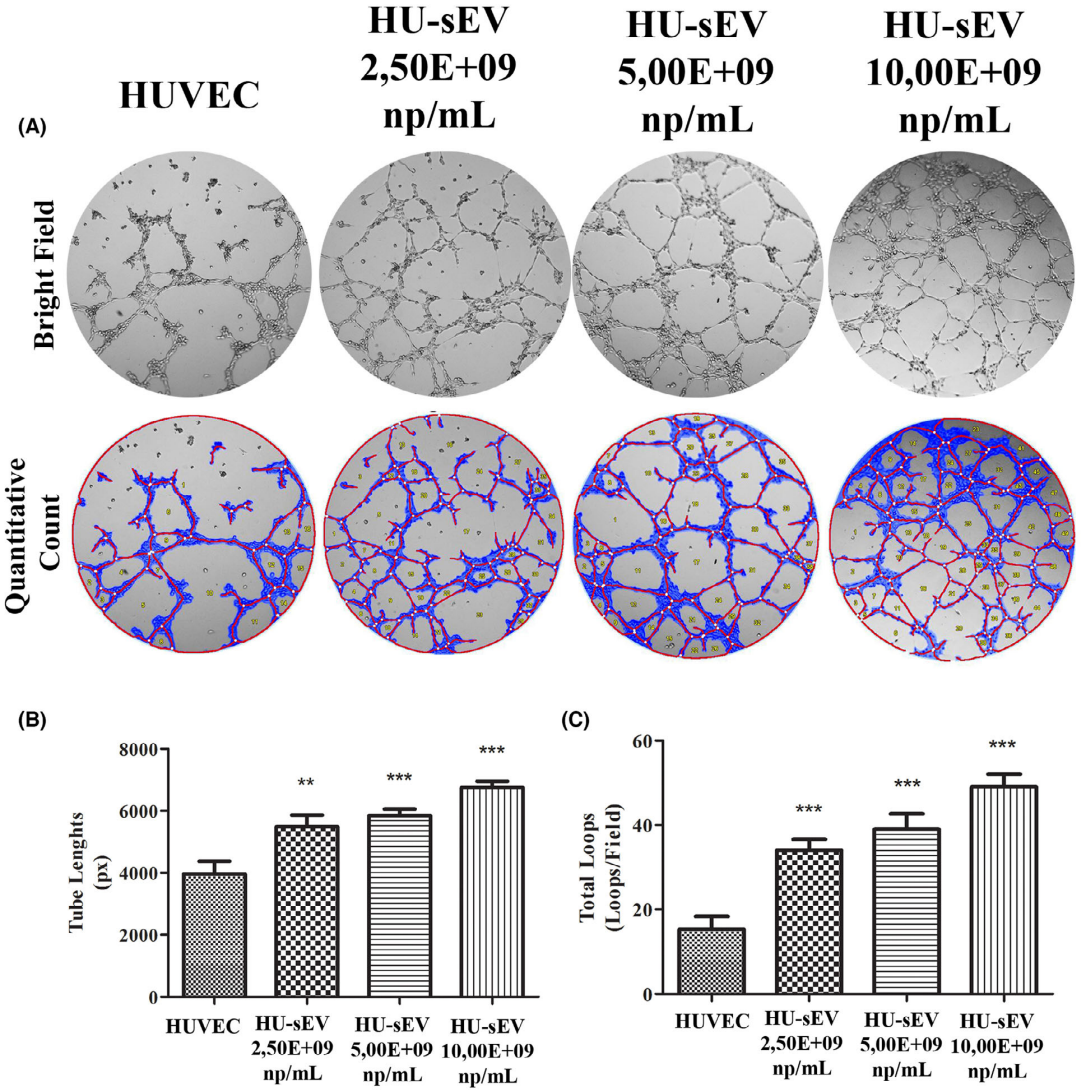
Tube formation of endothelial cells induced by three different concentrations of HU‐sEV for 7 h. (A) qualitative analysis evaluated by bright field 10× magnification light microscope images and quantitative analysis conducted by using Wimasis wimtube software. (B) Tube length and (C) loop values are also obtained from wimtube Software. The data were statistically analyzed using one‐way ANOVA with Tukey *post hoc* test. The data were presented as mean values ± SD. *n* = 3. **P < 0.01 and ***P < 0.001.

Tube formation assay was used to determine the effects of different concentrations of the HU‐sEVs on angiogenesis capacities of the HUVECs and MSCs (Fig. 7 and Fig. S1). All used concentrations of the HU‐sEVs significantly increased the tube lengths (Fig. 7A,B) and total loop number (Fig. 7A,C) compared with the control group. These effects were observed in a concentration‐dependent manner, where the highest concentration of the HU‐sEV gave the best result. When the HU‐sEV were used on the MSCs in tube formation assay, only the highest concentration of the HU‐sEV displayed significantly positive effect in terms of tube lengths (Fig. 7A,B) and total loops (Fig. 7A,C) compared with the control group.

### HU‐sEVs induce endothelial differentiation of MSCs

**Fig. 8:**
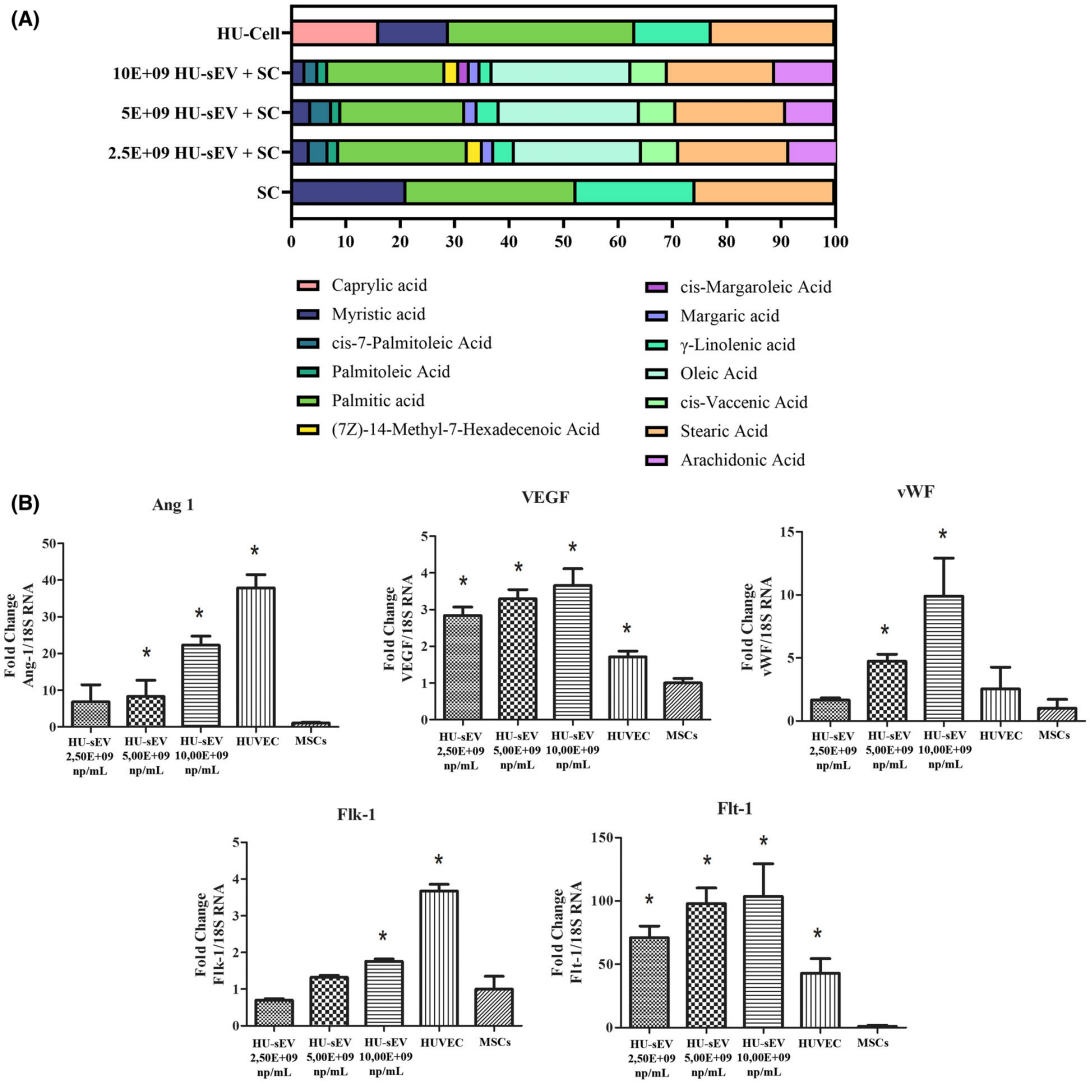
Effects of different dose of HU‐sEV treatment on (A) fatty acid profile and (B) gene expression levels of angiogenesis markers in MSCs as non‐differentiated negative control group and HUVEC as angiogenic positive control group *P < 0.05. (HUVEC, human embryonic vascular endothelial cells as positive vascular control; MSC, mesenchymal stem cells as untreated control). The data were statistically analyzed using one‐way ANOVA with Tukey *post hoc* test. The data were presented as mean values ± SEM. *n* = 3.

At the end of the differentiation protocol, 2.50E+09, 5.00E+09, and 10.00E+09 np·μL^−1^ of HU‐sEV treated groups, non‐treated MSC and HUVEC cells were collected from culture. The fatty acid profile of HU‐sEV treated MSCs and the expression levels of angiogenesis‐related genes such as Ang1, VEGF, Flt1, Flk1, and vWF were evaluated (Fig. 8). Non‐treated MSCs were used as a NC group, and HUVEC were used as a positive control group. In the results, it was observed that the fatty acid composition of MSCs altered noticeably in the result of HU‐sEVs treatment (Fig. 8A). Accordingly, newly emerged fatty acids like oleic acid, cis‐vaccenic acid, and arachidonic acid were observed in the fatty acid composition of EV‐treated MSCs. Besides, these increasing fatty acids were not observed in the fatty acid profile of HUVECs. Furthermore, caprylic acid, one of the fatty acids in HUVECs, could not be observed in the EV‐treated MSCs. On the contrary, as a result of HU‐sEV treatment, the percentages of myristic acid and γ‐linolenic acid decreased in the MSCs and so began resembling the fatty acid composition of HUVECs. In the gene expression analyses, all data were calculated and expressed as the fold change according to the NC group. While the highest expression level of the Ang1 gene was observed in the PC group (37.85 ± 3.66), its expression level was significantly increased in the 2.50E+09, 5.00E+09, and 10.00E+09 np·μL^−1^ groups compared with the NC group to 6.83 ± 4.65, 8.32 ± 4.4, and 22.3 ± 2.4, respectively. Besides, administration of the 2.50E+09 (2.84 ± 0.23), 5.00E+09 (3.29 ± 0.25), and 10.00E+09 (3.65 ± 0.25) np·μL^−1^ HU‐sEV upregulated VEGF levels compared with the PC (1.71 ± 0.15) and NC groups. Flk‐1 expression levels were significantly increased in the PC group (3.67 ± 0.17) compared with all other groups and just the highest particle number of HU‐sEV (1.75 ± 0.15) caused significant upregulation of the Flk‐1 expression compared with NC group. Flt‐1 expression levels were markedly upregulated, when the cells were exposed to the 2.50E+09 (70.89 ± 9.23), 5.00E+09 (97.85 ± 12.26), and 10.00E+09 (103.6 ± 25.7) np·μL^−1^ HU‐sEV compared with the PC (42.85 ± 11.57) and NC group. 5.00E+09 and 10.00E+09 np·μL^−1^ HU‐sEV caused overexpression of the vWF to 4.74 ± 0.54 and 9.90 ± 3.02, respectively, compared with the PC (2.54 ± 1.72) and NC groups (Fig. 8B).

## Discussion

Continuation of normal angiogenic activities is necessary for the health of individuals. In healthy individuals, a balance of inducing and suppressing factors keeps angiogenic activities at an equilibrium [25]. Failure in maintaining this balance can lead to fatal complications such as cerebral ischemia—the absence of nutrients and oxygen through the blood to the brain tissue [26].

Induction of angiogenesis in the infarct region acts as a natural defense mechanism as a result of the transport of nutrients and oxygen to the ischemic tissue [27]. It was determined that an increased density of capillaries in the brain was a factor that prolonged the life span of ischemia patients correlated with the enhancement in angiogenesis activities [28]. In a 2016 study, the preventive application of a herbal mixture called MLC901 before ischemic damage to mice improved their survival rates by inducing angiogenesis, leading to reductions in infarct areas [29], with other *in vivo* studies reporting similar results [29, 30]. Thus, it is clear that the increment of angiogenesis activities after the stroke leads to positive results in postischemia recovery.

Maintaining angiogenic activities without interruptions or alterations is necessary for the healthy being of individuals. Therefore, the determination of whether the angiogenesis activities occurring are normal or pathological is of the utmost importance. A balance of inducing and suppressing factors keeps angiogenic activities at an equilibrium [31]. Failures in maintaining this balance can lead to fatal complications such as cerebral ischemia, characterized by the absence of nutrients and oxygen through the blood to the brain tissue [32]. Therefore, it is very important to regulate angiogenesis positively at the beginning of the healing process of patients who recover from an ischemic crisis.

Induction of angiogenesis in the infarct region acts as a natural defense mechanism as a result of the transport of nutrients and oxygen to the ischemic tissue [27]. Besides, it was determined that an increased density of capillaries in the brain was a factor that prolonged the life span of ischemia patients correlated with the enhancement in angiogenesis activities [28]. In a 2016 study, the herbal mixture called MLC901 was applied as preventive purposes for 5 weeks before ischemic damage to mice [29]. In the recovery process after the ischemic damage, it was observed that survival rates of MLC901‐treated mice increased and the cerebral infarct regions decreased. Also, it has been determined that herbal treatment increases the proliferation of brain endothelial cells in the infarct area and effectuates the neovascular structures. Also, it was determined that similar results were obtained in other *in vivo* studies [29, 30]. Thus, it is clear that the increment of angiogenesis activities after the stroke leads to positive results in postischemia recovery.

However, when this equilibrium state slips on one side, angiogenesis‐related diseases begin to emerge. The most known examples of these diseases are ischemia, chronic inflammation, and cancer. These sorts of diseases are usually treated by inducing neovascular restoration due to the decrease in angiogenesis activities that result from the downregulation of pro‐angiogenic factors [31, 32].

Besides, a new vessel formation is an important point that has to be solved for tissue engineering applications. Even if all procedures necessary for regeneration are successfully carried out, an incomplete angiogenesis—and thus inability to provide adequate oxygen and nutrients—may cause a failure in tissue regeneration.

Small EVs (sEV) are nano‐sized vesicles secreted by all cell types that carry cellular cargo between cells. Composition of an EVs cargo depends on their cell‐of‐origin and the current physiological state of the cell, allowing them to take part in a variety of physiological functions [33]. Their unique nature has led to a field of rapidly expanding research, employing EVs as potential therapeutics [34], diagnosis of diseases [35], and delivery of drugs and nucleic acids [36]. HU‐sEV were positively characterized according to their surface markers. Positive presence of transmembrane proteins such as CD9, CD63, and CD81 supports the presence of a lipid membrane, while Alix, TSG101, and HSP70 supports the presence cytoplasmic cargo, which in tandem supports the presence of intact EVs in our samples. Western blots of CD9, CD81, and flotillin confirmed the presence of these markers in HU‐sEVs, while the negative GM130 and serum albumin results established that HU‐sEVs samples were not contaminated by co‐isolating proteins [24].

While researchers' study with stem cells to investigate the pro‐differentiation, pro‐angiogenic or pro‐migratory effects of any agent, the first thing to be done is the determination of sub‐lethal doses of the agent on the cells [37]. We observed that all applied groups of the HU‐sEV did not cause any significant effect on cell viability. Thus, the three lowest doses of the HU‐sEV were chosen to evaluate the pro‐angiogenic effects of them in a dose‐dependent manner. Besides, during endothelial differentiation of the MSCs, the cells gain capillary tube‐like structure and intercellular spaces occur. Administrations of sEV also caused formation of this structure in a dose‐dependent manner.

Gene Ontology enrichment of HU‐sEVs revealed a high degree of enrichment in proteins related to tube formation. The majority of HU‐sEVs proteins had functions in cytoskeleton organization, and the presence of proteins related to biological processes related to tube formation, such as migration or morphogenesis, were enriched >100 and 43.6‐fold, respectively, compared with the reference background. HU‐sEVs may induce angiogenesis and tube formation *in vivo* through these proteins. This is further supported by the fact that the majority of identified proteins were annotated as showing structural molecular activity. As expected, a large percentage of the identified proteins was annotated as of EV origin. Proteins that would be present if the EV population was contaminated by cell death, such as of nuclear or mitochondrial origin, were absent in the EV proteome.

When we compare the fatty acid contents of the cell and the exosome, the ratios of saturated fatty acid and unsaturated fatty acid do not differ significantly, but the ratios in the content of FAs change with an individual examination. While FAs with polyunsaturated fatty acid properties, which increase fluidity in particular, disappear in the exosome, the proportion of FAs that provide fluidity does not change with the emergence of branched fatty acids in the exosome when they are not present in the cell. Although there is no exosomal FAME analysis data that we can compare in the literature, the data obtained from our fatty acid analyzes have shown that the lipid profiles of exosomes are more saturated and more stable. Although this feature gives a more robust structure to exosomes, it has reduced its use as a drug‐loading system. According to the general profile of HU‐sEV, fluidity did not decrease and the saturated FAs did not increase, providing it with a more flexible structure. Although the decrease in fluidity reduces the half‐life of the EV, it has increased the possibility of loading drugs, nucleic acids, and similar substances into the EV. And this feature has given the EV the ability to be used as a carrier system.

Tube formation assay is a model experiment for evaluating angiogenesis capacity of the cells [38]. HUVECs, which are model cells for angiogenesis studies, were used. In addition, MSCs have angiogenesis capacity [39]. Matrigel has a unique chemical composition to induce angiogenesis of the cells and measurements of the total length and loop indicate angiogenesis capacity of the cells under certain conditions like sEV administration [40] in Matrigel assay. We observed that while angiogenesis capacity of the HUVEC is more than MSCs according to integrity of the tube structure, sEV treatment displayed different effects onto the cells. All particle numbers of the sEV increased total tube length and loop in HUVEC; however, only the highest particle number of the sEV caused an increase in MSCs.

When evaluating the differentiation capacity of cells, changes in the cell membrane of MSC cells can be a criterion for angiogenicity. The change in the FA ratios formed on the cell membrane by different doses of HU‐sEVs given on MSCs gives information about the differentiation of cells. In particular, the presence of oleic and cis‐vaccenic unsaturated fatty acids in MSCs treated with Hu‐sEV increases the fluidity of the cell membrane, and this feature facilitates the cells to gain vascularization properties and to change the cell structure more easily.

To trigger the vascular maturation *in vitro*, VEGF and Ang‐1 exhibit positive roles [41]. Besides, VEGF has interactions such as Flt1 and Flk1 [42, 43, 44]. The von Willebrand factor's main role is hemostasis, which plays a critical role in blood vessel formation [10, 11]. The highest particle numbers of the HU‐sEV increased all these critical gene expressions, while other applications caused partial changes in the related gene expressions. HUVECs displayed a pro‐angiogenic effect in a dose‐dependent manner in the aspect of the gene expression levels.

In conclusion, small EVs isolated from HUVEC cells induce pro‐angiogenic responses in MSCs. Treatment with HU‐sEVs increased the viability and tube‐forming potential of MSCs. These could be the result of the HU‐sEVs inducing the increased expression of pro‐angiogenic genes in treated MSCs. The pro‐angiogenic activity of the sEV was also confirmed on the HUVECs in a tube formation assay. According to these results, HU‐sEVs are promising candidates for the treatment of vascular diseases and wound healing studies. However, to reinforce this claim, the efficacy of the HU‐sEVs should also be tested *in vivo*.

As a future aspect, increasing the neovascularization potential of these EVs and triggering the cells in the environment for vascularization will lead to the use of these EVs in many fields, especially tissue engineering. In tissue engineering‐oriented studies, when it is desired to repair the damaged tissue or to create a new 3D tissue, even if the target tissue can be created, the inability to induce vascularization causes the tissue to deteriorate because the tissue cannot be fed properly. In addition, HU‐EVs have high biocompatibility and biodegradable properties that can be used both in local applications and in systems that can be mixed with scaffold and can be used as cell‐free therapy.

## Conflict of interest

The authors declare no conflict of interest.

## Author contributions

PNT, HA, and FŞ performed the experimental design of this study and the construction and analysis of the experiments. HA, PNT, OKK, and BTB performed cell culture, media collection, exosome isolation, and characterization. HA, OKK, BTB, TBH, and EAA performed molecular experiments and tube formation assay. PNT, EAA, and TBH structured the preparation of the figures. BTB, EAA, FŞ, HA, and OKK performed grammar correction and final writing. All authors read and approved the final manuscript.

## Supporting information


**Figure S1.** Tube formation of MSC induced by three different concentrations of HU‐sEV for 7 h. (a) qualitative analysis evaluated by bright field 10X magnification light microscope images and quantitative analysis conducted by using Wimasis WimTube software. (b) Tube length and (c) loop values are also obtained from WimTube Software. The data were statistically analyzed using one‐way ANOVA with Tukey post hoc test. The data were presented as mean values ± SD. n = 3. *p < 0.05 and ** p < 0.01.
**Table S1.** HU‐sEV proteins
**Table S2.** KEGG software analysis.

## Data Availability

The data that support the findings of this study are available from the corresponding author, P. Neslihan Taşlı, upon reasonable request.
