# Supplementary material for: Endothelial cell‐derived extracellular vesicles induce pro‐angiogenic responses in mesenchymal stem cells
Source: FEBS Open Bio. 2024 Mar 29;14(5):740–55. doi: 10.1002/2211-5463.13650 (PMC11073499; doi:10.1002/2211-5463.13650)
Supplement: Supplementary file 1 — Figure S1. Tube formation of MSC induced by three different concentrations of HU‐sEV for 7 h. (a) qualitative analysis evaluated by bright field 10X magnification light microscope images and quantitative analysis conducted by using Wimasis WimTube software. (b) Tube length and (c) loop values are also obtained from WimTube Software. The data were statistically analyzed using one‐way ANOVA with Tukey post hoc test. The data were presented as mean values ± SD. n = 3. *p < 0.05 and ** p < 0.01. Table S1. HU‐sEV proteins Table S2. KEGG software analysis. [file FEB4-14-740-s001.docx]

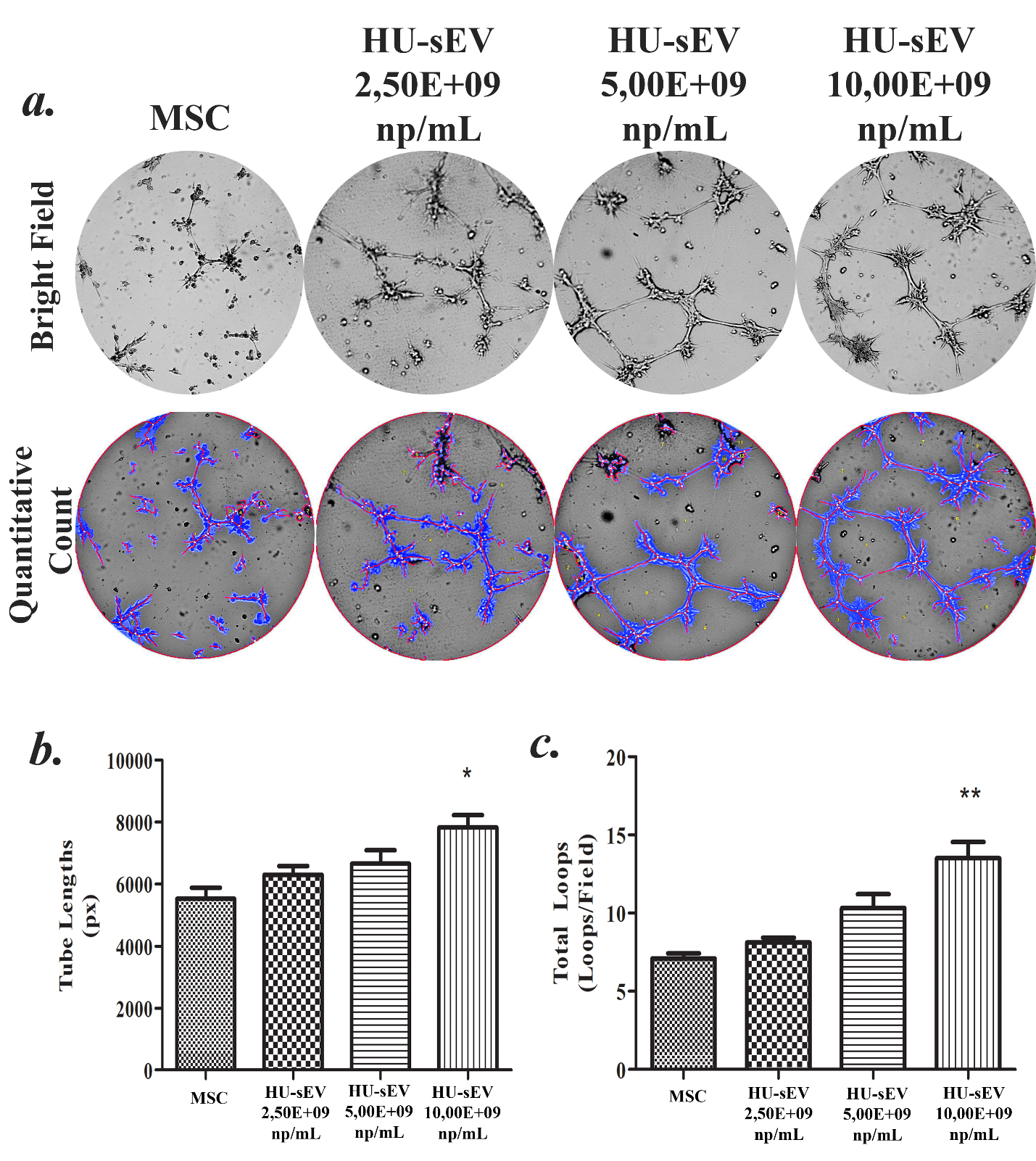


**Supplement Figure 1: Tube formation of MSC induced by three different concentrations of HU-sEV for 7 hours.** (a) qualitative analysis evaluated by bright field 10X magnification light microscope images and quantitative analysis conducted by using Wimasis WimTube software. (b) Tube length and (c) loop values are also obtained from WimTube Software. The data were statistically analyzed using one-way ANOVA with Tukey post-hoc test. The data were presented as mean values ± SD. n=3. *p < 0.05 and ** p <0.01.

**Supplement Table 1: Proteins identified in HU-sEV**

| Accession | Description |
| --- | --- |
| Q5THR3 | EF-hand calcium-binding domain-containing protein 6 OS=Homo sapiens GN=EFCAB6 PE=1 SV=1 |
| F5H5D3 | Tubulin alpha-1C chain OS=Homo sapiens GN=TUBA1C PE=1 SV=1 |
| P06733 | Alpha-enolase OS=Homo sapiens GN=ENO1 PE=1 SV=2 |
| Q14473 | Beta-globin gene from a thalassemia patient OS=Homo sapiens PE=3 SV=1 |
| P35908 | Keratin, type II cytoskeletal 2 epidermal OS=Homo sapiens GN=KRT2 PE=1 SV=2 |
| P19827 | Inter-alpha-trypsin inhibitor heavy chain H1 OS=Homo sapiens GN=ITIH1 PE=1 SV=3 |
| P81605 | Dermcidin OS=Homo sapiens GN=DCD PE=1 SV=2 |
| E7EQ64 | Trypsin-1 OS=Homo sapiens GN=PRSS1 PE=3 SV=1 |
| P02533 | Keratin, type I cytoskeletal 14 OS=Homo sapiens GN=KRT14 PE=1 SV=4 |
| B4DPP6 | cDNA FLJ54371, highly similar to Serum albumin OS=Homo sapiens PE=2 SV=1 |
| A0A024R035 | Complement component 9, isoform CRA_a OS=Homo sapiens GN=C9 PE=4 SV=1 |
| D3YTG3 | Target of Nesh-SH3 OS=Homo sapiens GN=ABI3BP PE=4 SV=1 |
| A0A024R9Q1 | Thrombospondin 1, isoform CRA_a OS=Homo sapiens GN=THBS1 PE=4 SV=1 |
| P13645 | Keratin, type I cytoskeletal 10 OS=Homo sapiens GN=KRT10 PE=1 SV=6 |
| Q9Y6V0 | Protein piccolo OS=Homo sapiens GN=PCLO PE=1 SV=4 |
| A0A024RCW6 | Peroxisome proliferative activated receptor, delta, isoform CRA_a OS=Homo sapiens GN=PPARD PE=3 SV=1 |
| A0A024R5Q9 | HCG1982563 OS=Homo sapiens GN=hCG_1982563 PE=4 SV=1 |
| A0A0A0MRJ7 | Coagulation factor V OS=Homo sapiens GN=F5 PE=4 SV=1 |
| A6NDB9 | Paralemmin-3 OS=Homo sapiens GN=PALM3 PE=1 SV=2 |
| P01023 | Alpha-2-macroglobulin OS=Homo sapiens GN=A2M PE=1 SV=3 |
| P06396 | Gelsolin OS=Homo sapiens GN=GSN PE=1 SV=1 |
| A0A024R944 | Serpin peptidase inhibitor, clade C (Antithrombin), member 1, isoform CRA_a OS=Homo sapiens GN=SERPINC1 PE=3 SV=1 |
| D3DRR6 | Inter-alpha (Globulin) inhibitor H2, isoform CRA_a OS=Homo sapiens GN=ITIH2 PE=4 SV=1 |
| J3KN16 | Proteasome-associated protein ECM29 homolog OS=Homo sapiens GN=KIAA0368 PE=1 SV=1 |
| M9PAN3 | MHC class I antigen (Fragment) OS=Homo sapiens GN=HLA-C PE=3 SV=1 |
| B7Z8Q2 | cDNA FLJ55606, highly similar to Alpha-2-HS-glycoprotein OS=Homo sapiens PE=2 SV=1 |
| Q6GMV7 | Uncharacterized protein OS=Homo sapiens PE=2 SV=1 |
| A5A3E0 | POTE ankyrin domain family member F OS=Homo sapiens GN=POTEF PE=1 SV=2 |
| H6VRG1 | Keratin 1 OS=Homo sapiens GN=KRT1 PE=3 SV=1 |
| P68366 | Tubulin alpha-4A chain OS=Homo sapiens GN=TUBA4A PE=1 SV=1 |
| P35527 | Keratin, type I cytoskeletal 9 OS=Homo sapiens GN=KRT9 PE=1 SV=3 |
| D1MGQ2 | Alpha-2 globin chain OS=Homo sapiens GN=HBA2 PE=3 SV=1 |
| P13647 | Keratin, type II cytoskeletal 5 OS=Homo sapiens GN=KRT5 PE=1 SV=3 |
| Q8WVW5 | Putative uncharacterized protein (Fragment) OS=Homo sapiens PE=2 SV=1 |
| A1E282 | Beta-actin (Fragment) OS=Homo sapiens PE=2 SV=1 |
| Q45KI0 | Trypsin I (Fragment) OS=Homo sapiens GN=PRSS1 PE=3 SV=1 |
| Q53G76 | Beta actin variant (Fragment) OS=Homo sapiens PE=2 SV=1 |
| B4DDU2 | cDNA FLJ60097, highly similar to Tubulin alpha-ubiquitous chain OS=Homo sapiens PE=2 SV=1 |
| Q52MT0 | Beta globin (Fragment) OS=Homo sapiens GN=HBB PE=3 SV=1 |
| Q5VYK3 | Proteasome-associated protein ECM29 homolog OS=Homo sapiens GN=ECM29 PE=1 SV=2 |
| B4E2Y1 | cDNA FLJ52879, highly similar to Peroxisome proliferator-activated receptordelta OS=Homo sapiens PE=2 SV=1 |
| Q9BQE3 | Tubulin alpha-1C chain OS=Homo sapiens GN=TUBA1C PE=1 SV=1 |
| Q86W18 | Protease serine 1 (Fragment) OS=Homo sapiens GN=PRSS1 PE=3 SV=1 |
| B3KUD3 | cDNA FLJ39583 fis, clone SKMUS2004897, highly similar to ACTIN, ALPHA SKELETAL MUSCLE OS=Homo sapiens PE=2 SV=1 |
| Q86W20 | Protease serine 1 (Fragment) OS=Homo sapiens GN=PRSS1 PE=3 SV=1 |
| F6KPG5 | Albumin (Fragment) OS=Homo sapiens PE=2 SV=1 |
| A0A087WTE1 | Inter-alpha-trypsin inhibitor heavy chain H2 OS=Homo sapiens GN=ITIH2 PE=1 SV=1 |
| D6RHD5 | Serum albumin OS=Homo sapiens GN=ALB PE=1 SV=1 |
| D1MGQ0 | Delta globin (Fragment) OS=Homo sapiens GN=HBD PE=3 SV=1 |
| A4D1A8 | Similar to Piccolo protein (Aczonin) OS=Homo sapiens GN=LOC392742 PE=4 SV=1 |
| B7Z2X4 | cDNA FLJ53327, highly similar to Gelsolin OS=Homo sapiens PE=2 SV=1 |
| Q5JPC9 | ABI gene family, member 3 (NESH) binding protein, isoform CRA_d OS=Homo sapiens GN=DKFZp667H216 PE=2 SV=1 |
| B3KXF2 | cDNA FLJ45314 fis, clone BRHIP3005142, highly similar to Proteasome-associated protein ECM29 homolog OS=Homo sapiens PE=2 SV=1 |
| C9JEV8 | Tubulin alpha-4A chain (Fragment) OS=Homo sapiens GN=TUBA4A PE=1 SV=1 |
| Q4ZGM8 | Hemoglobin alpha-2 globin mutant (Fragment) OS=Homo sapiens PE=3 SV=1 |
| H0Y8D1 | Trypsin-1 (Fragment) OS=Homo sapiens GN=PRSS1 PE=3 SV=1 |
| S6BAP8 | IgG L chain OS=Homo sapiens PE=2 SV=1 |
| Q9GZL9 | Beta-globin (Fragment) OS=Homo sapiens GN=HBB PE=3 SV=1 |
| H0YA55 | Serum albumin (Fragment) OS=Homo sapiens GN=ALB PE=1 SV=1 |
| A0A087WYN7 | Immunoglobulin lambda-like polypeptide 5 OS=Homo sapiens GN=IGLL5 PE=4 SV=1 |
| Q71U36 | Tubulin alpha-1A chain OS=Homo sapiens GN=TUBA1A PE=1 SV=1 |
| H7C4S3 | Target of Nesh-SH3 (Fragment) OS=Homo sapiens GN=ABI3BP PE=4 SV=4 |
| B4DPR2 | cDNA FLJ50830, highly similar to Serum albumin OS=Homo sapiens PE=2 SV=1 |
| P02748 | Complement component C9 OS=Homo sapiens GN=C9 PE=1 SV=2 |
| B8ZZJ2 | Actin, gamma-enteric smooth muscle OS=Homo sapiens GN=ACTG2 PE=3 SV=1 |
| A2RTY6 | Inter-alpha (Globulin) inhibitor H2 OS=Homo sapiens GN=ITIH2 PE=2 SV=1 |
| Q86W19 | Protease serine 1 (Fragment) OS=Homo sapiens GN=PRSS1 PE=3 SV=1 |
| A6NL76 | Actin, alpha skeletal muscle OS=Homo sapiens GN=ACTA1 PE=3 SV=3 |
| B7Z6I1 | cDNA FLJ52755, highly similar to Actin, aortic smooth muscle OS=Homo sapiens PE=2 SV=1 |
| Q1KLZ0 | HCG15971, isoform CRA_a OS=Homo sapiens GN=PS1TP5BP1 PE=2 SV=1 |
| A0A087WWT3 | Serum albumin OS=Homo sapiens GN=ALB PE=1 SV=1 |
| A0A087WYR4 | Immunoglobulin lambda-like polypeptide 5 OS=Homo sapiens GN=IGLL5 PE=4 SV=1 |
| B2M1S8 | Delta-globin B2 variant (Fragment) OS=Homo sapiens GN=HBD PE=3 SV=1 |
| Q5T8M8 | Actin, alpha skeletal muscle OS=Homo sapiens GN=ACTA1 PE=3 SV=1 |
| Q6GMW4 | IGL@ protein OS=Homo sapiens GN=IGL@ PE=2 SV=1 |
| B4E335 | cDNA FLJ52842, highly similar to Actin, cytoplasmic 1 OS=Homo sapiens PE=2 SV=1 |
| A4UCS8 | Enolase (Fragment) OS=Homo sapiens PE=2 SV=1 |
| F6UVQ4 | Actin, aortic smooth muscle (Fragment) OS=Homo sapiens GN=ACTA2 PE=1 SV=1 |
| Q4JLR8 | Hemoglobin beta chain (Fragment) OS=Homo sapiens GN=HBB PE=3 SV=1 |
| B4E3A4 | cDNA FLJ57283, highly similar to Actin, cytoplasmic 2 OS=Homo sapiens PE=2 SV=1 |
| Q6S8J3 | POTE ankyrin domain family member E OS=Homo sapiens GN=POTEE PE=1 SV=3 |
| P68032 | Actin, alpha cardiac muscle 1 OS=Homo sapiens GN=ACTC1 PE=1 SV=1 |
| L0R5C4 | Alternative protein POTEM OS=Homo sapiens GN=POTEM PE=3 SV=1 |
| B4DQK4 | cDNA FLJ53743, highly similar to Tubulin alpha-3 chain OS=Homo sapiens PE=2 SV=1 |
| A1YZ08 | Beta-globin (Fragment) OS=Homo sapiens GN=HBB PE=3 SV=1 |
| Q670S4 | Hemoglobin Lepore-Baltimore (Fragment) OS=Homo sapiens PE=3 SV=1 |
| B3KPS3 | cDNA FLJ32131 fis, clone PEBLM2000267, highly similar to Tubulin alpha-ubiquitous chain OS=Homo sapiens PE=2 SV=1 |
| A0A087WX49 | Immunoglobulin lambda-like polypeptide 5 OS=Homo sapiens GN=IGLL5 PE=4 SV=1 |
| A0PJG0 | THBS1 protein (Fragment) OS=Homo sapiens GN=THBS1 PE=2 SV=1 |
| B3KW67 | cDNA FLJ42347 fis, clone UTERU2003399, highly similar to Actin, gamma-enteric smooth muscle OS=Homo sapiens PE=2 SV=1 |
| P63261 | Actin, cytoplasmic 2 OS=Homo sapiens GN=ACTG1 PE=1 SV=1 |
| I3L3R2 | Actin, cytoplasmic 2 (Fragment) OS=Homo sapiens GN=ACTG1 PE=1 SV=1 |
| C9JZR7 | Actin, cytoplasmic 1 (Fragment) OS=Homo sapiens GN=ACTB PE=1 SV=4 |
| B4DW52 | cDNA FLJ55253, highly similar to Actin, cytoplasmic 1 OS=Homo sapiens PE=2 SV=1 |
| F8WAS2 | Inter-alpha-trypsin inhibitor heavy chain H1 OS=Homo sapiens GN=ITIH1 PE=4 SV=1 |
| A0A087WWC9 | Immunoglobulin lambda-like polypeptide 5 OS=Homo sapiens GN=IGLL5 PE=4 SV=1 |
| Q8N532 | TUBA1C protein OS=Homo sapiens GN=TUBA1C PE=2 SV=1 |
| Q53ZX7 | Protease serine 1 (Fragment) OS=Homo sapiens GN=PRSS1 PE=3 SV=1 |
| Q6GMW6 | Uncharacterized protein OS=Homo sapiens PE=2 SV=1 |
| B7WNR0 | Serum albumin OS=Homo sapiens GN=ALB PE=1 SV=1 |
| A0A0A0MS51 | Gelsolin OS=Homo sapiens GN=GSN PE=4 SV=1 |
| C9JDL2 | Tubulin alpha-4A chain (Fragment) OS=Homo sapiens GN=TUBA4A PE=1 SV=4 |
| Q6J1Z7 | Hemoglobin beta (Fragment) OS=Homo sapiens GN=HBB PE=3 SV=1 |
| B4DSV9 | cDNA FLJ56632, moderately similar to Target of Nesh-SH3 OS=Homo sapiens PE=2 SV=1 |
| P0CG38 | POTE ankyrin domain family member I OS=Homo sapiens GN=POTEI PE=3 SV=1 |
| S6C4Q9 | IgG L chain OS=Homo sapiens PE=2 SV=1 |
| Q5T0H8 | Gelsolin OS=Homo sapiens GN=GSN PE=1 SV=1 |
| Q96E61 | Uncharacterized protein OS=Homo sapiens PE=2 SV=1 |
| P68363 | Tubulin alpha-1B chain OS=Homo sapiens GN=TUBA1B PE=1 SV=1 |
| B7Z9A0 | cDNA FLJ56212, highly similar to Gelsolin OS=Homo sapiens PE=2 SV=1 |
| C9JKR2 | Albumin, isoform CRA_k OS=Homo sapiens GN=ALB PE=4 SV=1 |
| P62736 | Actin, aortic smooth muscle OS=Homo sapiens GN=ACTA2 PE=1 SV=1 |
| Q4F786 | Hemoglobin delta Etolia variant (Fragment) OS=Homo sapiens GN=HBD PE=3 SV=1 |
| Q7Z7J6 | Actin alpha 1 skeletal muscle protein OS=Homo sapiens GN=ACTA1 PE=2 SV=1 |
| B4DVQ0 | cDNA FLJ58286, highly similar to Actin, cytoplasmic 2 OS=Homo sapiens PE=2 SV=1 |
| P68133 | Actin, alpha skeletal muscle OS=Homo sapiens GN=ACTA1 PE=1 SV=1 |
| A8K9P0 | cDNA FLJ78413, highly similar to Homo sapiens albumin, mRNA OS=Homo sapiens PE=2 SV=1 |
| B3VL86 | Mutant beta-globin OS=Homo sapiens GN=HBB PE=3 SV=1 |
| Q5T8M7 | Actin, alpha skeletal muscle OS=Homo sapiens GN=ACTA1 PE=3 SV=1 |
| I3L1U9 | Actin, cytoplasmic 2 (Fragment) OS=Homo sapiens GN=ACTG1 PE=1 SV=1 |
| C9JTX5 | Actin, cytoplasmic 1 (Fragment) OS=Homo sapiens GN=ACTB PE=1 SV=1 |
| F8WCH0 | Actin, gamma-enteric smooth muscle OS=Homo sapiens GN=ACTG2 PE=4 SV=1 |
| K7EM38 | Actin, cytoplasmic 2 (Fragment) OS=Homo sapiens GN=ACTG1 PE=1 SV=1 |
| Q5T0H9 | Gelsolin OS=Homo sapiens GN=GSN PE=1 SV=1 |
| C9JQ00 | Tubulin alpha-4A chain (Fragment) OS=Homo sapiens GN=TUBA4A PE=1 SV=1 |
| Q5T985 | Inter-alpha-trypsin inhibitor heavy chain H2 OS=Homo sapiens GN=ITIH2 PE=1 SV=1 |
| I3L4N8 | Actin, cytoplasmic 2 (Fragment) OS=Homo sapiens GN=ACTG1 PE=1 SV=4 |
| Q6P5S3 | Uncharacterized protein OS=Homo sapiens PE=2 SV=1 |
| C9JFL5 | Actin, gamma-enteric smooth muscle (Fragment) OS=Homo sapiens GN=ACTG2 PE=3 SV=4 |
| Q13707 | ACTA2 protein (Fragment) OS=Homo sapiens GN=ACTA2 PE=3 SV=1 |
| A6XGL3 | Protease serine 1 OS=Homo sapiens GN=PRSS1 PE=2 SV=1 |
| A0A087WXC3 | Immunoglobulin lambda-like polypeptide 5 OS=Homo sapiens GN=IGLL5 PE=4 SV=1 |
| F8WB63 | Actin, gamma-enteric smooth muscle OS=Homo sapiens GN=ACTG2 PE=3 SV=1 |
| Q4TWB7 | Beta globin (Fragment) OS=Homo sapiens GN=HBB PE=3 SV=1 |
| P0CG39 | POTE ankyrin domain family member J OS=Homo sapiens GN=POTEJ PE=3 SV=1 |
| Q68D69 | Putative uncharacterized protein DKFZp779G1236 OS=Homo sapiens GN=DKFZp779G1236 PE=2 SV=1 |
| Q9BWU5 | Mutant hemoglobin beta chain (Fragment) OS=Homo sapiens GN=HBB PE=3 SV=1 |
| F1D8S7 | Peroxisome proliferative activated receptor, delta, isoform CRA_b OS=Homo sapiens GN=NR1C2 PE=2 SV=1 |
| P02765 | Alpha-2-HS-glycoprotein OS=Homo sapiens GN=AHSG PE=1 SV=1 |
| K7EM90 | Enolase (Fragment) OS=Homo sapiens GN=ENO1 PE=1 SV=1 |
| P07996 | Thrombospondin-1 OS=Homo sapiens GN=THBS1 PE=1 SV=2 |
| B2RBS8 | cDNA, FLJ95666, highly similar to Homo sapiens albumin (ALB), mRNA OS=Homo sapiens PE=2 SV=1 |
| E9M4D4 | Hemoglobin alpha-1 globin chain (Fragment) OS=Homo sapiens GN=HBA1 PE=3 SV=1 |
| E9PE96 | Protein piccolo OS=Homo sapiens GN=PCLO PE=4 SV=1 |
| Q6NS95 | IGL@ protein OS=Homo sapiens GN=IGL@ PE=2 SV=1 |
| E9NGZ5 | Hemoglobin beta globin chain (Fragment) OS=Homo sapiens GN=HBB PE=3 SV=1 |
| Q53GA7 | Tubulin alpha 6 variant (Fragment) OS=Homo sapiens PE=2 SV=1 |
| A0A5E4 | Uncharacterized protein OS=Homo sapiens PE=2 SV=1 |
| A0N071 | Delta globin OS=Homo sapiens GN=HBD PE=3 SV=1 |
| P04264 | Keratin, type II cytoskeletal 1 OS=Homo sapiens GN=KRT1 PE=1 SV=6 |
| B7Z539 | cDNA FLJ56954, highly similar to Inter-alpha-trypsin inhibitor heavy chain H1 OS=Homo sapiens PE=2 SV=1 |
| Q8IZI0 | Hemoglobin beta chain variant Hb-I_Toulouse (Fragment) OS=Homo sapiens GN=HBB PE=3 SV=1 |
| A0A0A0MT01 | Gelsolin OS=Homo sapiens GN=GSN PE=4 SV=1 |
| C9JJQ8 | Tubulin alpha-4A chain (Fragment) OS=Homo sapiens GN=TUBA4A PE=1 SV=1 |
| Q96T46 | Hemoglobin alpha 2 (Fragment) OS=Homo sapiens GN=HBA2 PE=3 SV=1 |
| Q5FWF9 | IGL@ protein OS=Homo sapiens GN=IGL@ PE=1 SV=1 |
| Q4TZM4 | Hemoglobin beta chain (Fragment) OS=Homo sapiens GN=HBB PE=3 SV=1 |
| A0A087WW55 | Trypsin-1 OS=Homo sapiens GN=PRSS1 PE=3 SV=1 |
| B3KS49 | cDNA FLJ35478 fis, clone SMINT2007796, highly similar to Gelsolin OS=Homo sapiens PE=2 SV=1 |
| H6VRF8 | Keratin 1 OS=Homo sapiens GN=KRT1 PE=3 SV=1 |
| B3VL05 | Beta globin (Fragment) OS=Homo sapiens PE=3 SV=1 |
| G3V1N2 | HCG1745306, isoform CRA_a OS=Homo sapiens GN=HBA2 PE=3 SV=1 |
| C8C504 | Beta-globin OS=Homo sapiens GN=HBB PE=3 SV=1 |
| I1VZV6 | Hemoglobin alpha 1 OS=Homo sapiens GN=HBA1 PE=3 SV=1 |
| H6VRG0 | Keratin 1 OS=Homo sapiens GN=KRT1 PE=3 SV=1 |
| Q5XTR9 | Hemoglobin delta-beta fusion protein (Fragment) OS=Homo sapiens GN=HBD/HBB PE=3 SV=1 |
| A1A508 | PRSS3 protein OS=Homo sapiens GN=PRSS3 PE=2 SV=1 |
| Q53ZX9 | Protease serine 1 (Fragment) OS=Homo sapiens GN=PRSS1 PE=3 SV=1 |
| A0A087WWT5 | Immunoglobulin lambda-like polypeptide 5 OS=Homo sapiens GN=IGLL5 PE=4 SV=1 |
| B7Z4U6 | cDNA FLJ55803, highly similar to Gelsolin OS=Homo sapiens PE=2 SV=1 |
| U6A216 | Mutant hemoglobin alpha 1 globin chain (Fragment) OS=Homo sapiens GN=HBA1 PE=3 SV=1 |
| P02768 | Serum albumin OS=Homo sapiens GN=ALB PE=1 SV=2 |
| Q56G89 | Serum albumin OS=Homo sapiens PE=2 SV=1 |
| Q8IUL9 | Hemoglobin beta chain variant Hb.Sinai-Bel Air (Fragment) OS=Homo sapiens GN=HBB PE=3 SV=1 |
| Q6PIK1 | IGL@ protein OS=Homo sapiens GN=IGL@ PE=1 SV=1 |
| C9JV77 | Alpha-2-HS-glycoprotein OS=Homo sapiens GN=AHSG PE=1 SV=1 |
| H0Y897 | Target of Nesh-SH3 (Fragment) OS=Homo sapiens GN=ABI3BP PE=4 SV=1 |
| A0A087WWU7 | Immunoglobulin lambda-like polypeptide 5 OS=Homo sapiens GN=IGLL5 PE=4 SV=1 |
| Q86W17 | Protease serine 1 (Fragment) OS=Homo sapiens GN=PRSS1 PE=3 SV=1 |
| E9PFT6 | Hemoglobin subunit delta OS=Homo sapiens GN=HBD PE=1 SV=1 |
| P63267 | Actin, gamma-enteric smooth muscle OS=Homo sapiens GN=ACTG2 PE=1 SV=1 |
| B2M1S6 | Beta-globin Tacoma variant (Fragment) OS=Homo sapiens GN=HBB PE=3 SV=1 |
| Q6PEY2 | Tubulin alpha-3E chain OS=Homo sapiens GN=TUBA3E PE=1 SV=2 |
| B4DM79 | cDNA FLJ53848, highly similar to Inter-alpha-trypsin inhibitor heavy chain H2 OS=Homo sapiens PE=2 SV=1 |
| P07477 | Trypsin-1 OS=Homo sapiens GN=PRSS1 PE=1 SV=1 |
| B9A064 | Immunoglobulin lambda-like polypeptide 5 OS=Homo sapiens GN=IGLL5 PE=2 SV=2 |
| G5E9R0 | Actin, cytoplasmic 1 OS=Homo sapiens GN=ACTB PE=3 SV=1 |
| A0A087X2A1 | Immunoglobulin lambda-like polypeptide 5 OS=Homo sapiens GN=IGLL5 PE=4 SV=1 |
| Q9UQM3 | Alpha-tubulin (Fragment) OS=Homo sapiens PE=2 SV=1 |
| Q3LR79 | Hemoglobin beta (Fragment) OS=Homo sapiens GN=HBB PE=3 SV=1 |
| A8K3K1 | cDNA FLJ78096, highly similar to Homo sapiens actin, alpha, cardiac muscle (ACTC), mRNA OS=Homo sapiens PE=2 SV=1 |
| P19823 | Inter-alpha-trypsin inhibitor heavy chain H2 OS=Homo sapiens GN=ITIH2 PE=1 SV=2 |
| C9JUM1 | Actin, cytoplasmic 1 (Fragment) OS=Homo sapiens GN=ACTB PE=1 SV=1 |
| Q0Z944 | Beta globin (Fragment) OS=Homo sapiens GN=HBB PE=3 SV=1 |
| P12259 | Coagulation factor V OS=Homo sapiens GN=F5 PE=1 SV=4 |
| H6VRG3 | Keratin 1 OS=Homo sapiens GN=KRT1 PE=3 SV=1 |
| Q9BWV6 | Mutant beta globin OS=Homo sapiens GN=HBB PE=3 SV=1 |
| E7EVS6 | Actin, cytoplasmic 1 (Fragment) OS=Homo sapiens GN=ACTB PE=1 SV=4 |
| Q53ZX8 | Protease serine 1 (Fragment) OS=Homo sapiens GN=PRSS1 PE=3 SV=1 |
| D9YZU5 | Hemoglobin, beta OS=Homo sapiens GN=HBB PE=3 SV=1 |
| A0A087WU42 | Immunoglobulin lambda-like polypeptide 5 OS=Homo sapiens GN=IGLL5 PE=4 SV=1 |
| Q6ZU41 | cDNA FLJ44011 fis, clone TESTI4024420, highly similar to Rattus norvegicus presynaptic cytomatrix protein (Pclo) OS=Homo sapiens PE=2 SV=1 |
| Q53G99 | Beta actin variant (Fragment) OS=Homo sapiens PE=2 SV=1 |
| Q6V0K9 | Mutant hemoglobin beta chain (Fragment) OS=Homo sapiens GN=HBB PE=3 SV=1 |
| Q3Y9I8 | Hemoglobin beta (Fragment) OS=Homo sapiens GN=HBB PE=3 SV=1 |
| Q53GK6 | Beta actin variant (Fragment) OS=Homo sapiens PE=2 SV=1 |
| B7Z992 | cDNA FLJ53698, highly similar to Gelsolin OS=Homo sapiens PE=2 SV=1 |
| B7Z549 | cDNA FLJ56821, highly similar to Inter-alpha-trypsin inhibitor heavy chain H1 OS=Homo sapiens PE=2 SV=1 |
| Q13748 | Tubulin alpha-3C/D chain OS=Homo sapiens GN=TUBA3C PE=1 SV=3 |
| B3KT06 | cDNA FLJ37398 fis, clone BRAMY2027467, highly similar to Tubulin alpha-ubiquitous chain OS=Homo sapiens PE=2 SV=1 |
| Q9UK54 | Hemoglobin beta subunit variant (Fragment) OS=Homo sapiens GN=HBB PE=2 SV=1 |
| J3KT65 | Actin, cytoplasmic 2 OS=Homo sapiens GN=ACTG1 PE=1 SV=1 |
| B7Z6P1 | cDNA FLJ53662, highly similar to Actin, alpha skeletal muscle OS=Homo sapiens PE=2 SV=1 |
| A8JZY9 | cDNA FLJ78587 OS=Homo sapiens PE=2 SV=1 |

**Supplement Table 2: Pathways associated with the KO Term**

| **Metabolism** |  |
| --- | --- |
|  |  |
| Global and overview maps |  |
| 01100 Metabolic pathways | 1 |
| 01110 Biosynthesis of secondary metabolites | 1 |
| 01120 Microbial metabolism in diverse environments | 1 |
| 01200 Carbon metabolism | 1 |
| 01230 Biosynthesis of amino acids | 1 |
| Carbohydrate metabolism |  |
| 00010 Glycolysis / Gluconeogenesis | 1 |
| Energy metabolism |  |
| 00680 Methane metabolism | 1 |
|  |  |
| **Genetic Information Processing** |  |
|  |  |
| Folding, sorting and degradation |  |
| 03018 RNA degradation | 1 |
|  |  |
| **Environmental Information Processing** |  |
|  |  |
| Signal transduction |  |
| 04015 Rap1 signaling pathway | 2 |
| 04310 Wnt signaling pathway | 1 |
| 04350 TGF-beta signaling pathway | 1 |
| 04390 Hippo signaling pathway | 1 |
| 04391 Hippo signaling pathway - fly | 1 |
| 04371 Apelin signaling pathway | 1 |
| 04066 HIF-1 signaling pathway | 1 |
| 04151 PI3K-Akt signaling pathway | 1 |
| Signaling molecules and interaction |  |
| 04080 Neuroactive ligand-receptor interaction | 1 |
| 04512 ECM-receptor interaction | 1 |
| 04514 Cell adhesion molecules | 1 |
|  |  |
| **Cellular Processes** |  |
|  |  |
| Transport and catabolism |  |
| 04144 Endocytosis | 1 |
| 04145 Phagosome | 4 |
| Cell growth and death |  |
| 04210 Apoptosis | 2 |
| 04115 p53 signaling pathway | 1 |
| 04218 Cellular senescence | 1 |
| Cellular community - eukaryotes |  |
| 04510 Focal adhesion | 2 |
| 04520 Adherens junction | 1 |
| 04530 Tight junction | 2 |
| 04540 Gap junction | 1 |
| Cell motility |  |
| 04810 Regulation of actin cytoskeleton | 2 |
|  |  |
| **Organismal Systems** |  |
|  |  |
| Immune system |  |
| 04610 Complement and coagulation cascades | 4 |
| 04611 Platelet activation | 1 |
| 04613 Neutrophil extracellular trap formation | 1 |
| 04612 Antigen processing and presentation | 1 |
| 04666 Fc gamma R-mediated phagocytosis | 1 |
| 04670 Leukocyte transendothelial migration | 1 |
| Endocrine system |  |
| 04911 Insulin secretion | 1 |
| 03320 PPAR signaling pathway | 1 |
| 04915 Estrogen signaling pathway | 1 |
| 04921 Oxytocin signaling pathway | 1 |
| 04926 Relaxin signaling pathway | 1 |
| 04918 Thyroid hormone synthesis | 1 |
| 04919 Thyroid hormone signaling pathway | 1 |
| Circulatory system |  |
| 04260 Cardiac muscle contraction | 1 |
| 04261 Adrenergic signaling in cardiomyocytes | 1 |
| 04270 Vascular smooth muscle contraction | 2 |
| Digestive system |  |
| 04971 Gastric acid secretion | 1 |
| 04972 Pancreatic secretion | 1 |
| 04974 Protein digestion and absorption | 1 |
| Sensory system |  |
| 04745 Phototransduction - fly | 1 |
| Environmental adaptation |  |
| 04714 Thermogenesis | 1 |
|  |  |
| **Human Diseases** |  |
|  |  |
| Cancer: overview |  |
| 05200 Pathways in cancer | 1 |
| 05206 MicroRNAs in cancer | 1 |
| 05205 Proteoglycans in cancer | 2 |
| 05203 Viral carcinogenesis | 2 |
| Cancer: specific types |  |
| 05225 Hepatocellular carcinoma | 1 |
| 05221 Acute myeloid leukemia | 1 |
| 05219 Bladder cancer | 1 |
| Infectious disease: viral |  |
| 05166 Human T-cell leukemia virus 1 infection | 1 |
| 05170 Human immunodeficiency virus 1 infection | 1 |
| 05171 Coronavirus disease - COVID-19 | 1 |
| 05164 Influenza A | 2 |
| 05168 Herpes simplex virus 1 infection | 1 |
| 05163 Human cytomegalovirus infection | 1 |
| 05167 Kaposi sarcoma-associated herpesvirus infection | 1 |
| 05169 Epstein-Barr virus infection | 1 |
| 05165 Human papillomavirus infection | 2 |
| Infectious disease: bacterial |  |
| 05110 Vibrio cholerae infection | 1 |
| 05130 Pathogenic Escherichia coli infection | 2 |
| 05132 Salmonella infection | 2 |
| 05131 Shigellosis | 1 |
| 05135 Yersinia infection | 1 |
| 05150 Staphylococcus aureus infection | 1 |
| 05100 Bacterial invasion of epithelial cells | 1 |
| Infectious disease: parasitic |  |
| 05146 Amoebiasis | 1 |
| 05144 Malaria | 3 |
| 05143 African trypanosomiasis | 2 |
| Immune disease |  |
| 05322 Systemic lupus erythematosus | 1 |
| 05320 Autoimmune thyroid disease | 1 |
| 05330 Allograft rejection | 1 |
| 05332 Graft-versus-host disease | 1 |
| 05340 Primary immunodeficiency | 1 |
| Neurodegenerative disease |  |
| 05010 Alzheimer disease | 1 |
| 05012 Parkinson disease | 1 |
| 05014 Amyotrophic lateral sclerosis | 2 |
| 05016 Huntington disease | 1 |
| 05020 Prion disease | 2 |
| 05022 Pathways of neurodegeneration - multiple diseases | 1 |
| Cardiovascular disease |  |
| 05418 Fluid shear stress and atherosclerosis | 1 |
| 05410 Hypertrophic cardiomyopathy | 2 |
| 05412 Arrhythmogenic right ventricular cardiomyopathy | 1 |
| 05414 Dilated cardiomyopathy | 2 |
| 05416 Viral myocarditis | 2 |
| Endocrine and metabolic disease |  |
| 04940 Type I diabetes mellitus | 1 |
